# High-performance pyrite nano-catalyst driven photothermal/chemodynamic synergistic therapy for Osteosarcoma

**DOI:** 10.1186/s12951-024-02419-2

**Published:** 2024-04-01

**Authors:** Meirong Li, Minghua Wang, Junfeng Huang, Shiqi Tang, Jingyu Yang, Zhourui Xu, Gaixia Xu, Xin Chen, Jia Liu, Chengbin Yang

**Affiliations:** 1 Central Laboratory, The Second Affiliated Hospital of the Chinese University of Hong Kong, Shenzhen & Longgang District People’s Hospital of Shenzhen, Shenzhen, Guangdong 518172 P. R. China; 2 Guangdong Key Laboratory for Biomedical Measurements and Ultrasound Imaging, School of Biomedical Engineering, Shenzhen University Medical School, Shenzhen University, Shenzhen, Guangdong 518060 P. R. China; 3 Pathology Department, The Second Affiliated Hospital of the Chinese University of Hong Kong, Shenzhen & Longgang District People’s Hospital of Shenzhen, Shenzhen, Guangdong 518172 P. R. China; 4 School of Mechanical Engineering, Dongguan University of Technology, Dongguan, Guangdong 523808 P. R. China

**Keywords:** Pyrite nanoparticle, Fenton catalyst, Photothermal therapy, Chemodynamic therapy, Osteosarcoma

## Abstract

Osteosarcoma (OS) is an aggressive bone tumor with strong invasiveness, rapid metastasis, and dreadful mortality. Chemotherapy is a commonly used approach for OS treatment but is limited by the development of drug resistance and long-term adverse effects. To date, OS still lacks the curative treatment. Herein, we fabricated pyrite-based nanoparticles (FeS_2_@CP NPs) as synergetic therapeutic platform by integrating photothermal therapy (PTT) and chemo-dynamic therapy (CDT) into one system. The synthetic FeS_2_@CP NPs showed superior Fenton reaction catalytic activity. FeS_2_@CP NPs-based CDT efficaciously eradicated the tumor cells by initiating dual-effect of killing of apoptosis and ferroptosis. Furthermore, the generated heat from FeS_2_@CP under near-infrared region II (NIR-II) laser irradiation could not only inhibit tumor’s growth, but also promote tumor cell apoptosis and ferroptosis by accelerating •OH production and GSH depletion. Finally, the photothermal/NIR II-enhanced CDT synergistic therapy showed excellent osteosarcoma treatment effects both in vitro and in vivo with negligible side effects. Overall, this work provided a high-performance and multifunctional Fenton catalyst for osteosarcoma synergistic therapy, which provided a pathway for the clinical application of PTT augmented CDT.

**Supplementary Information:**

The online version contains supplementary material available at 10.1186/s12951-024-02419-2.

## Introduction

Osteosarcoma (OS) is a primary malignant bone tumor, which is one of the most common malignant tumors in young adults and children [1]. OS can cause disability and death in children and adolescents due to its strong invasive activity, rapid metastasis, high recurrence rate, and poor prognosis [2]. The common therapeutic strategies of OS are surgery and adjuvant chemotherapy, while unsatisfactory therapeutic effects, long-term toxic side effects, and drug-resistance seriously jeopardize the quality of a patient’s life [3, 4]. Currently, the 5-year survival rate of patients with localized osteosarcoma is about 60%. When OS patients are diagnosed with metastatic disease, the 5-year survival rate rapidly drops to approximately 20% [3]. Even worse, treated patients continue to suffer from secondary tumors and recurrence. Therefore, it is urgent to pursue a novel strategy to address the above issues and improve the survival of OS patients.

Photothermal therapy (PTT) can utilize heat converted from light energy to kill tumor cells [5], which has been recognized as a next-generation therapeutic strategy of OS. The good biocompatibility and high photothermal conversion efficiency (PCE) of the photothermal agent (PTA) are the primary considerations of PTT in clinical application [6]. Besides, the limited tissue penetration depth of the near-infrared (NIR) laser seriously hinders the clinical translation of PTT. The NIR-II laser (1000–1700 nm) is expected to replace the conventional NIR-I laser (700–900 nm) as the trigger to improve the penetration depth of PTT [7]. Because of the abnormal metabolism of the tumor, the tumor microenvironment (TME) has some unique characteristics, such as hypoxia, overproduced hydrogen peroxide (H_2_O_2_), and acidic matrix [8]. The fabrication of TME-responsive theranostic agents is extremely promising to overcome the challenge of cancer therapy. Benefiting from the overproduced hydrogen peroxide (H_2_O_2_), nano-catalysts can be utilized to generate high cytotoxic hydroxyl radical (•OH) from H_2_O_2_ [8]. Through catalyzing the Fenton or Fenton-like reactions, the produced •OH could be elaborately used as chemodynamic therapy (CDT) agent, which selectively kill the tumors cell, while not disturbing normal tissues [9]. The H_2_O_2_ content and suitable acid conditions (pH = 2.0 ∼ 3.0) are the main rate-limiting factors of Fenton reaction [10]. However, insufficient acidity (pH 6.5–6.9) and limited H_2_O_2_ content (typically 0.1-1 mM) in TME decrease the •OH production rate, thus jeopardizing the therapeutic efficiency of CDT [9]. Therefore, it is urgent to develop high-performance Fenton catalysts for generating sufficient •OH to eradicate tumor cells. In addition, many effective nanotechnology-based tumor therapy strategies, such as PTT, have been performed to combine CDT [11]. The Fenton reaction efficiency and •OH productivity can be significantly enhanced when the temperature of the tumor site rises [12]. Therefore, a suitable combination of PTT and CDT is expected to synergistically overcome the limitations of monotherapy and improve the tumor therapeutic effect.

Ferroptosis is an iron-dependent non-apoptotic cell death. Lipid-hydrolytic peroxides (LPO) accumulate in the ferroptosis process when the cell membrane lipids are irreversibly oxidized by reactive oxygen species (ROS) [13]. Ferroptosis can be activated in tumor cells by increasing the production of •OH through raised iron concentration. In addition, lipid hydroperoxidase glutathione peroxidase 4 (GPX4) uses glutathione (GSH) as a co-factor to degrade reactive LPO to inert lipid alcohols, thereby preventing the formation of ROS [14]. The reduction of highly toxic lipid ROS can effectively prevent cell membrane damage and cell ferroptosis. Thus, GPX4 inactivation caused by GSH depletion can also induce ferroptosis for tumor treatment [13, 14]. Iron-based nanomaterials have strong oxidation ability, and their variable valence state endows them with excellent catalytic ability to produce ROS via the Fenton reaction [25, 28]. CDT mainly depends on the production of •OH from the Fenton reaction to induce tumor cell ferroptosis [15]. The accumulated intracellular ROS can oxidize mitochondrial cardiolipin and activate the Bax/Bak cell signal pathway [16]. Then, a series of pro-apoptotic proteins are subsequently generated, resulting in cell death through the mitochondrial apoptosis pathway [17]. Intracellular GSH may be depleted as a result of the ROS activity in the Fenton reaction [18, 19]. The depletion of GSH can improve the therapeutic efficiency of ROS-based therapy by activating cell apoptosis or ferroptosis [20]. In addition, iron-based materials also display photothermal characteristics under NIR laser irradiation, which could not only directly ablate the tumor but also accelerate Fenton reactivity in the tumor CDT synergetic treatment [9]. And in the process of NIR II-enhanced CDT, ferroptosis is further promoted with a significantly increased intracellular LPO level [21]. Taken together, iron-based materials have great potential to combine PTT and NIR-enhanced CDT to drive apoptosis and ferroptosis for highly efficient tumor therapy.

**Scheme 1:**
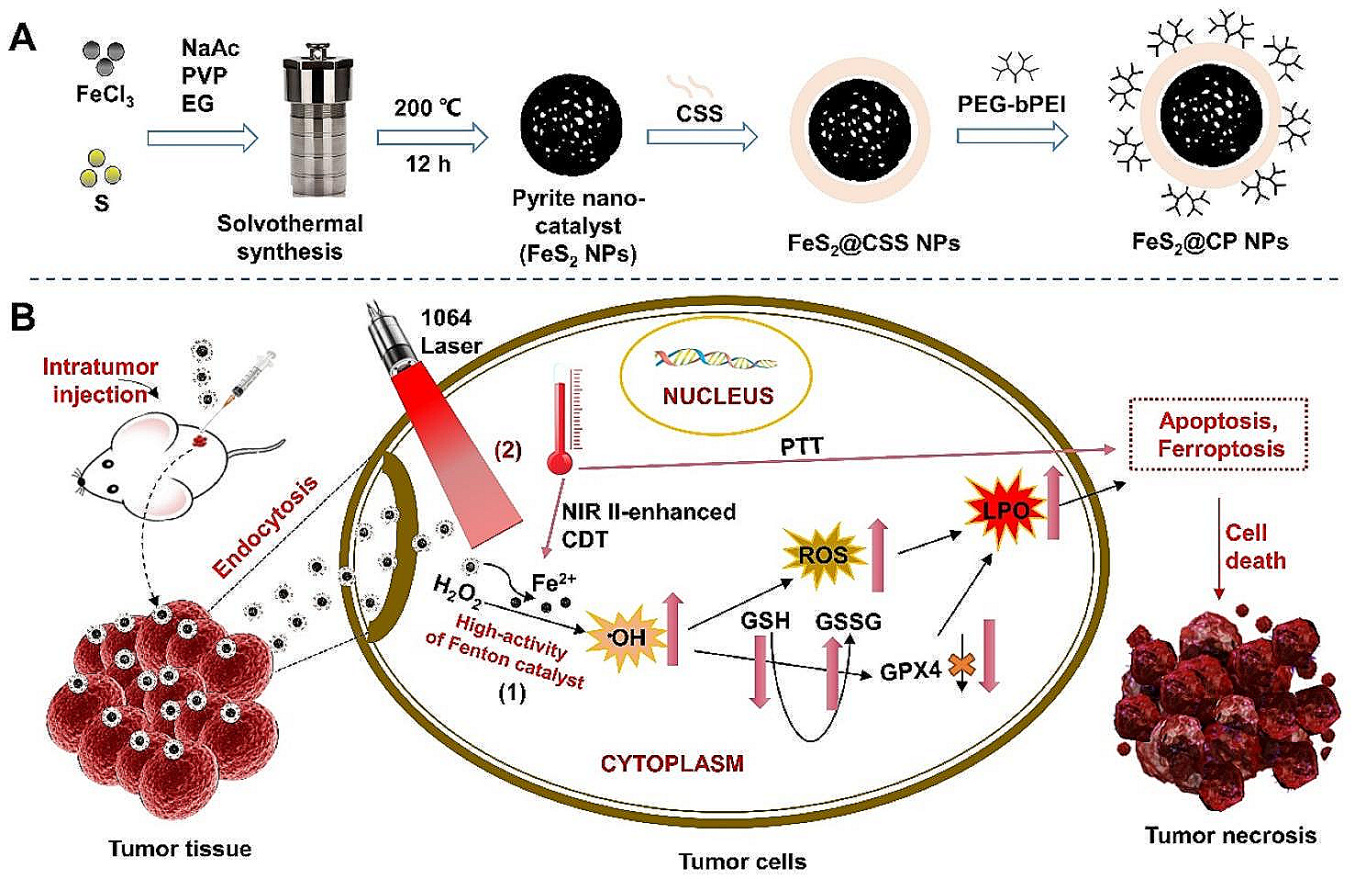
Schematic illustration of the preparation of FeS_2_-based nanoparticles (pyrite-based NPs) and their roles in NIR II-enhanced CDT synergistic therapy of osteosarcoma. (**A**) The fabrication process of FeS_2_@CP NPs. (**B**) The therapeutic mechanism of FeS_2_@CP NPs in the treatment of osteosarcoma

As a Fenton catalyst, Pyrite (FeS_2_) nanomaterials exhibit great enzymatic ability, narrow band gap, and high theoretical capacity [22]. Herein, a high-performance Fenton catalyst, pyrite NPs, was successfully fabricated and characterized, and then modified with carboxymethyl-dextran (CMD) sodium salt (CSS) and polyethylene glycol-branched polyetherimide (PEG-bPEI), and named as FeS_2_@CP NPs. The functionalized FeS_2_@CP NPs were introduced to treat osteosarcoma in vitro and in vivo by activating an apoptosis-ferroptosis dual-killing mechanism (Scheme 1). FeS_2_@CP NPs show biodegradability, low cytotoxicity, and high catalytic activity. The limited H_2_O_2_ in TME was efficiently catalyzed by FeS_2_@CP NPs to form •OH. The produced •OH was expected to cause LPO and intracellular GSH depletion, then GXP4 would be inactivated, and ultimately induced apoptosis and ferroptosis of tumor cells. More importantly, depending on the high PCE and good biocompatibility, FeS_2_@CP NPs exhibit an excellent NIR II-enhanced nano-catalytic synergistic anticancer effect. In this study, the developed synergistic anticancer strategy based on FeS_2_@CP NPs-driven PTT and CDT provides a promising approach for the treatment of OS.

## Materials and methods

### Synthesis of FeS_2_ NPs

FeS_2_ NPs were synthesized by the one-pot method according to the process described in reference [23, 24] with a slight modification. The detailed strategy was described as follows and shown in the schematic illustration. Typically, 0.5 g of polyvinylpyrrolidone (PVP, MW:10,000; Cat. No. PVP10, Sigma-Aldrich, USA) was dissolved uniformly into 30 mL ethylene glycol (EG; Cat. No. 875,310, Macklin, Shanghai, China) solution with ultrasonication for 20 min, 0.3 g iron chloride anhydrous (FeCl_3_; Cat. No. 157,740, Macklin, Shanghai, China) was then added with continuous ultrasonication for 20 min. Subsequently, 3.6 g sodium acetate (NaAc•3H_2_O; Cat. No. 71,188, Sigma Aldrich, USA) and 0.4 g sulfur powder (S; Cat. No. 043766, Alfa Aesar, UK) were successively added to the mixture under constant magnetic stirring for 20 min each. The resultant homogeneous mixture was then heated and maintained at 200 °C for 12 h in a 50 mL autoclave. The black precipitants were collected by centrifugation at 10 000 rpm for 10 min and washed with chloroform, ethanol absolute and ultra-pure water three times successively to remove the excess sulfur powder and impurities. Finally, the products were dried in a vacuum freeze-dryer overnight to collect pyrite nanoparticles (FeS_2_ NPs) for further characterization and use.

### The fabrication of FeS_2_@CP NPs

The amount of 1 mg lyophilized pyrite (FeS_2_) NPs powder was dissolved uniformly into 1 mL ultra-pure water. Then, 50 µL aqueous solution of carboxymethyl-dextran (CMD) sodium salt (CSS, 100 mg/mL; Cat. No. R053005, Ron reagents, Shanghai, China) was added, and the mixture was under constant magnetic stirring overnight. The intermediate product of FeS_2_ coated with CSS (shorthand for FeS_2_@CSS NPs) was collected by centrifugation at 10,000 rpm for 5 min. After washing with ultra-pure water twice, FeS_2_@CSS NPs were dissolved uniformly into 1 mL of ultra-pure water. Then 50 µL of polyethylene glycol-branched polyetherimide (PEG-bPEI, 100 mg/mL; Cat. No. 900,926, Sigma Aldrich, USA) was added. After similar overnight stirring, centrifugation, and washing steps, the precipitate product of FeS_2_@CSS coated with PEG-bPEI (shorthand for FeS_2_@CP NPs) was obtained. In the 2-step preparation process, the final mass ratio of FeS_2_ NPs to CSS or to PEG-bPEI in the mixture was both 5:1. Finally, the modified pyrite-based NPs were resuspended in water or PBS buffer and stored at 4 °C for further use in biological experiments.

In order to obtain tracer capability, FeS_2_-based NPs were further labeled with Cy3-PEG-SH. Briefly, Cy3-PEG-SH (2 mg/mL; Cat. No. 410,201, Delta-f, Xi’an, China.) was added into a FeS_2_-based NPs (1 mg/mL) chloroform suspension, and then the suspension was stirred overnight and followed with centrifugation at 10,000 rpm for 10 min. The precipitation was washed with ethanol absolute and ultra-pure water three times successively to remove the excess Cy3-PEG-SH by centrifugation at 4 °C and 10,000 rpm for 10 min. Finally, the Cy3-labeled-pyrite-based NPs (Cy3-FeS_2_ NPs and Cy3-FeS_2_@CP NPs) were resuspended in water or PBS buffer and stored at 4 °C for biological experiment use.

### Characterization of the formulated NPs

The morphologies of the FeS_2_-based NPs were characterized by transmission electron microscope (TEM) (HT7700, Hitachi, Japan) and scanning electron microscope (SEM) (S4800, Hitachi, Japan). The microstructure and compositional distribution of the nanocrystals were further investigated by selected area electron diffraction (SAED) pattern and EDX-elemental mapping (Energy Dispersive X-Ray Spectroscopy) by using a high-resolution transmission electron microscope (HTEM; JEM-F200, JEOL, Japan). The chemical state and composition of the NPs were characterized by X-ray photoelectron spectroscopy (XPS) (ESCALAB250Xi, Thermo Fisher, USA). X-ray diffraction spectrum (XRD) was obtained by a D8Advance X-ray diffractometer (Bruker, Germany). Absorbance spectra of the nanoparticle samples were recorded by using a UV-Vis-NIR spectrophotometer (Tianjin Tuopu Instrument, TP-720). The hydrodynamic diameter and Zeta potential (surface potential) values of FeS_2_-based NPs with or without modification were measured by dynamic light scattering (DLS) (Nano-ZS90, Malvern, UK) to verify the dispersion and stability of these nanoparticles.

### Evaluation of photothermal effect

The photothermal character evaluation of FeS_2_-based NPs was carried out by a thermal imaging system containing a 1064 nm laser and a FLIR A300 infrared thermal imaging camera. For in vitro concentration-dependent evaluation, laser (1 W/cm^2^) illuminated on a 1.5 mL centrifuge tube containing 1 mL of FeS_2_ NPs with different concentrations (0, 12.5, 25, 50, 100, 200, and 400 µg/mL). During the 10-minute observation period, a thermal camera was used to record the temperature alteration with an accuracy of 0.1 °C. Accordingly, tubes containing 1 mL of 50 µg/mL or 100 µg/mL FeS_2_ NPs were exposed to the 1064 nm laser irradiation with different power densities (0.5, 1.0, 1.5, and 2.0 W/cm^2^) for 10 min to obtain the temperature alteration. The photothermal stability of FeS_2_ NPs (50 µg/mL or 100 µg/mL) suspension was measured by a continuous cycle (three times) of 10 min 1 W/cm^2^ irradiation and natural cooling to room temperature.

### The calculation of photothermal conversion efficiency

To evaluate the photothermal conversion efficiency (PCE) of FeS_2_ NPs, the suspension of NPs was irradiated by laser (1064 nm, 1 W/cm^2^) for 10 min to obtain saturation temperature and then cooled down to room temperature with the laser turned off. The PCE (η) can be calculated by using Eqs. 1–4 as follows:

1 $$\eta = {\text{ }}\left[ {hS\left( {{T_{max}} - {T_{ssur}}} \right){\text{ }} - {Q_{dis}}} \right]{\text{ }}/I\left( {1 - {{10}^{ - A1064}}} \right)$$

2 $${\tau _s}_ = {m_D}{C_D}/hS$$

3 $$t = {\text{ }} - {\tau _s}ln\theta$$

4 $$\theta = {\text{ }}\left( {T - {T_{ssur}}} \right){\text{ }}/{\text{ }}\left( {{T_{max}} - {T_{ssur}}} \right)$$

Where “*h*” is the heat transfer coefficient, “*S*” is the surface area of the container, and the value of “*hS*” can be obtained according to the cooling curve. *T*_*max*_ is the steady-state temperature of photothermal agents, while *T*_*ssur*_ is the surrounding temperature. *Q*_*dis*_ represents the energy absorbed by the container and solvent. “*I*” is the power of incident laser power (1 W/cm^2^), while *A*_*1064*_ is the absorbance of photothermal agents at 1064 nm. *m*_D_ represents the mass of photothermal agents. *C*_*D*_ is the specific heat capacity of the solvent, and which of water is 4.2 × 10^3^ J/(kg•℃). *t* is the time between turning off the laser and naturally cooling to room temperature. *T* represents the temperature at time *t*.

### Confirmation of •OH production

Hydroxyphenyl fluorescein (HPF) can only be oxidized by highly reactive oxygen species (hROS), such as •OH and peroxynitrite, to produce strong green fluorescence (Ex/Em, ∼ 490/515 nm) [25]. Therefore, HPF can be used to determine the production of·•OH when H_2_O_2_ is used as substrate. In brief, 20 µL of FeS_2_-based NPs with different final concentrations were added into 1960 µL of Na_2_HPO_4_-citrate buffer (pH 4.5) containing 20 µL of H_2_O_2_ (100 mM). After incubation at room temperature for 10 min, the mixture was centrifuged at 10,000 rpm for 5 min. Then 1.5 µL HPF (5 mM in dimethyl sulfoxide: DMSO) was added to 1.5 mL of the supernatant. After incubation for 15 min in the dark, the fluorescence intensity between 500 and 600 nm was detected by a fluorescence photometer (Lumina Pro, Thermo scientific, USA). Constant volume Na_2_HPO_4_-citrate buffer for baseline determination (Blank group: Blk).

### Catalytic activity detection and kinetic assay

The catalytic activity of FeS_2_-based NPs as Fenton reagent was assessed using 3,3′,5,5′-tetramethylbenzidine (TMB; No. 613,548, Sigma-Aldrich, USA) as the substrate in the presence of H_2_O_2_. Briefly, 10 µL of different concentrations of FeS_2_-based NPs were added into the mixture containing 80 µL of Na_2_HPO_4_-citrate buffer (pH 4.5), 5 µL of TMB (20 mg/mL in DMSO) and 5 µL of H_2_O_2_ (10 mM). The final concentrations of NPs were 0, 1.25, 2.5, 5, 10, 20, and 40 µg/mL, respectively. After the reaction at room temperature (RT) for 10 min, the UV-vis absorbance spectra at 652 nm of oxidized TMB were recorded with a micro-plate reader (EPOCH-2, BioTek, USA). pH dependence assay of catalytic activity was carried out at RT for 10 min with a final concentration of 20 µg/mL NPs in different pH buffers (2.2, 2.5, 3.0, 3.5, 4.0, 4.5, 5.0, 5.5, 6.0, 6.5, 7.0, 7.5, 8.0). Temperature dependence experiments of catalytic activity were performed for 10 min with a final concentration of 20 µg/mL NPs in pH 4.5 buffer at different temperatures (0, 4, 25, 30, 35, 40, 45, 50, 55 °C).

For the steady-state kinetic assays of FeS_2_-based NPs with H_2_O_2_ as the substrate, 5 µL of different concentrations of H_2_O_2_ were added into the mixture containing 80 µL of Na_2_HPO_4_-citrate buffer (pH 4.5), 5 µL of TMB (20 mg/mL in DMSO) and 10 µL of FeS_2_-based NPs (0.2 mg/mL). The reaction was carried out for 10 min at 37 ℃. The final concentrations of H_2_O_2_ were 0, 2.5, 5, 10, 20, 40, 80, 160, 320 mM, respectively. The laser treatment groups were supplemented with laser irradiation (1064 nm, 1 W/cm^2^) during the 10 min reaction period under the same experimental conditions as above.

In the kinetic assay, the Michaelis constant (*K*_*M*_) is defined as the substrate concentration at half the maximal reaction velocity. *K*_*M*_ reflects the affinity of the catalyst to the substrate, and the smaller the K, the higher the affinity. The maximum reaction rate (*V*_*max*_) is the maximal reaction velocity that is observed at saturating substrate concentrations. The kinetics constants *K*_*M*_ and *V*_*max*_ were calculated by GraphPad Prism 7.0 software by fitting the initial reaction velocity values (*v*) and the substrate concentrations [S] to the Michaelis-Menten equation (Eq. 5):

5 $$v = {\text{ }}\left( {{V_{max}} \times {\text{ }}\left[ S \right]} \right){\text{ }}/{\text{ }}\left( {{K_M} + {\text{ }}\left[ S \right]} \right)$$

Where *v* is the initial reaction velocity, the calculation formula is Eq. 6:

6 $$v = {\text{ }}\Delta A/{\text{ }}\left( {\Delta t \times l \times \varepsilon } \right)$$

Where Δ*A* means the change of absorbance value, Δ*t* is the reaction time (s). *ε* is the molar absorption coefficient of the colorimetric substrate, which is generally 39 000 M^− 1^ cm^− 1^ for oxidized TMB at 652 nm. *l* is the path length of light traveling in the cuvette (cm).

The catalytic constant (*k*_*cat*_) is defined as the maximum number of substrate molecules converted to product per unit of time and calculated as the following Eq. 7:

7 $${k_{cat}} = {V_{max}}/\left[ E \right]$$

Where [E] is the concentration of the catalyst (M), which has to go from mass concentration to quantity concentration to molar concentration (the amount of substance concentration) for nanoparticles, correlated with the substantial weight density, mass concentration, and average volume for nanoparticles. In the conversion process, the mass density (*ρ)* of pyrite FeS_2_ is 5.02 g/L, the average nanometer diameter is 175 nm, the volume of the nanoparticle is calculated according to the spherical volume formula V= (4 / 3) πr³, and Avogadro constant is 6.022 × 10²³.

### Hemolytic assay

The hemolysis assay was employed to investigate the biocompatibility of FeS_2_-based NPs on red blood cells (RBCs). 5 mL of PBS (pH 7.4) was added to a 15 mL anticoagulant tube with 5 mL of anticoagulant blood from healthy Balb/c mice. The mixture was mixed gently and centrifuged at 4 °C and 3000 rpm for 15 min. Then the supernatant was discarded and the precipitated RBCs were washed three times with 10 mL PBS. The final working solution for the hemolysis assay consisted of 5% (v/v) of RBCs in PBS. To evaluate the hemolytic effect, 800 µL of FeS_2_-based NPs at various final concentrations (5, 10, 20, 40, 50, 80, 100, 160, 200 µg/mL) were mixed gently with 200 µL RBCs working solution in a 1.5 mL anticoagulant tube. Ultra-pure water and PBS were used as a positive control (PC) and negative control (NC), respectively. After incubation at room temperature for 3 h, the mixture was centrifuged at 4 °C and 3000 rpm for 5 min. The centrifuged product was photographed. Then 100 µL of the supernatants was extracted in a 96-well plate for quantifying the hemoglobin by measuring the absorbance at 540 nm under a microplate reader. The hemolysis rate was calculated as follows: Hemolysis rate (%) = (A_540_ of sample – A_540_ of NC) / (A_540_ of PC − A_540_ of NC) × 100%.

### In vitro degradation assay

In order to evaluate whether FeS_2_-based NPs can be biodegraded in a near-physiological environment, NPs were incubated in PBS buffer under pH 4.5 and pH 7.4, which mimicked the lysosome and internal recycle environment, respectively. At different incubation time points of 1, 7, and 15 days at room temperature, samples were taken out and observed by TEM.

### Hematotoxicity and hepatotoxicity evaluation

Hematotoxicity and hepatotoxicity evaluation were carried out to further monitor the in vivo biosafety of FeS_2_-based NPs. Six-week-old female Balb/c mice were randomly divided into three groups and received one intravenous (i.v.) injection of 100 µL of PBS buffer, FeS_2_ NPs and FeS_2_@CP NPs, respectively. The dose of pyrite-based NPs was 60 mg/kg, and the mass referred to the nanoparticles based on FeS_2_. The body weight curve of mice was recorded every 3 days during the 15-day monitoring with the observation of eating, drinking, and physical behavior. At the endpoint of monitoring, anticoagulant blood samples were collected in tubes with EDTA-Na_2_, and clotted blood samples were obtained for serum collection. Finally, mice were sacrificed by overdose of isoflurane to collect main organs (heart, liver, spleen, kidney, lung) for organ index calculation and histomorphological observation via H&E staining. Organ index = organ weight (mg) / body weight (g). An automatic hematology analyzer (RJ-0C107223, Mindray, Shenzhen, China) was used to detect the blood routine values of anticoagulant blood samples. And a blood biochemical analyzer (BS-220, Mindray, Shenzhen, China) was used to detect the biochemical analysis of serum blood samples according to the manufacturer’s instructions.

### Cell culture

We procured human osteosarcoma cell lines U2-OS (ATCC® HTB-96™, USA) and MNNG-HOS (ATCC® CRL-1547™, USA) from the National Collection of Authenticated Cell Cultures in Shanghai, China. The human umbilical vein endothelial cell line HUCVE (No. C0035C, Gibco, USA) was obtained from Thermo Fisher Scientific. The culture medium for MNNG-HOS and HUCVE cells was Dulbecco’s Modified Eagle Medium (DMEM; No.11,965,092, Gibco, USA) enriched with 10% fetal bovine serum (FBS; No.16,140,071, Gibco, USA), whereas U2-OS cells were cultured in Minimum Essential Medium (MEM; No.11,095,080, Gibco, USA) with the same FBS concentration. All cell lines were incubated at 37 °C in a humidified atmosphere containing 5% CO_2_.

### Mice and feeding

Five to six weeks old female Balb/c mice or athymic Balb/c nu/nu mice were purchased from the Medical Laboratory Animal Center of Guangdong Province, China. All mice were kept in individually ventilated cages (IVCs) of a specific pathogen freedom (SPF) environment, accompanied by a standard 12-hour light/dark cycle and ad libitum access to food and water. All animal experiments were approved by the Animal Ethical and Welfare Committee of Shenzhen University (AEWC-SZU) with the approval number AEWC-202,200,013 and were performed strictly according to animal care guidelines.

### Transfection efficiency detection

The amount of 1 × 10^5^ cells per well of osteosarcoma cells was seeded and cultured in the 12-well plate overnight for adherence. After replacing the complete medium with 500 µL Opti-MEM, cells were transfected with the Cy3-labeled- FeS_2_-based NPs for 1 and 6 h, respectively. The final concentration of NPs was 20 µg/mL. After transfection, the cells were washed with PBS and digested with 0.25% Trypsin-EDTA solution (Cat. No. T1300, Solarbio, Shanghai, China). Finally, the intracellular transfection efficiency of Cy3-labeled-FeS_2_-based NPs was detected by flow cytometry (FCM, CytoFLEX, Beckman, USA) with the channel set for Cy3. Meanwhile, the mean fluorescence intensity (MFI) recorded in FCM detection was also analyzed.

### Cellular Uptaken and subcellular distribution

The amount of 2 × 10^5^ cells seeded in the confocal dish for overnight culture was transfected with the Cy3-labeled FeS_2_-based NPs for 1 and 6 h, respectively. The final concentration of NPs was 20 µg/mL. After transfection, cells were washed three times with PBS. Then, cells were stained with 1 mL preheated PBS with 50 nM Lyso-Tracker Green (DND 26, Cat. No. C1047, Beyotime, China) and 0.01 mg/mL Hoechst 33,342 (Cat. NO. 62,249, Thermo Scientific, USA) for 30 min at 37 °C. Next, the staining buffer was removed, and cells were washed three times with PBS. Finally, the cells were immersed in 1 mL HBS for endo-lysosome escape observation by using a confocal laser scanning microscope (CLSM, ZEISS LSM880 AiryScan, Jena, Germany) with the filters set for DAPI, FAM, and Cy3.

### Intracellular reactive oxygen species (ROS) assay

Referring to the above 24-well plate transfection and irradiation conditions with FeS_2_-based NPs, the treated cells were cultured for 24 h. Then, digested cells were washed twice with PBS and stained via a ROS Assay Kit (No. S0033, Beyotime, China) with the fluorescence probe 2′,7′-dichlorofluorescin diacetate (DCFH-DA) for 20 min at 37 °C according to the manufacturer’s instructions. Rosup was used as a positive control drug. After twice washing, cells were detected by flow cytometry with the channel set at FITC for intracellular ROS assay. Meanwhile, the MFI of the FITC channel recorded in FCM detection was also analyzed.

### Intracellular lipid peroxidation (LPO) measurement

Referring to the above 24-well plate transfection condition and irradiation conditions with FeS_2_-based NPs, the treated cells were cultured for 24 h. Then, digested cells were washed twice with PBS and stained with C11-BODIPY 581/591 probe (Cat. No. 27,086, Cayman Chemical, USA) for 20 min at 37 °C according to the manufacturer’s instructions. After twice washing, cells were detected by flow cytometry with the channel set at FITC for cellular lipid peroxidation (LPO) measurement. Meanwhile, the MFI of the FITC channel recorded in FCM detection was also analyzed.

### Intracellular GSH and GSSG content assay

Referring to the above 24-well plate transfection and irradiation conditions with FeS_2−_based NPs, the treated cells were cultured for 24 h. Then, digested cells were washed twice with PBS. The cellular reduced glutathione (GSH) and oxidized glutathione disulfide (GSSG) content were measured with a GSH and GSSG Assay Kit (Cat. No. S0053, Beyotime, China) using the single point assay method according to the manufacturer’s instructions. The absorbance at 412 nm (A_412_) was measured by a microplate reader. The ratio of GSH/total glutathione and the rate of GSH/GSSG were calculated to assess the depletion of intracellular GSH in osteosarcoma cells by experimental treatment, respectively. Note: GSH = Total Glutathione – 2 × GSSG.

### Cell apoptosis assay

The amount of 5 × 10^4^ cells per well was seeded in a 24-well plate and cultured overnight for adherence. After being transfected with different concentrations of FeS_2_-based NPs (20, 40, 60 µg/mL) for 1 h, cells were treated with or without laser (1064 nm, 1 W/cm^2^) for 10 min. Then, cells were cultured for another 24 h and collected by trypsin. After being washed twice with cold PBS, cells were suspended in 100 µL 1 × binding buffer and stained with Annexin V-FITC/PI Apoptosis Detection Kit (Cat. No. 556,570, BD Pharmingen, USA) for 15 min in the dark at room temperature. Finally, the number of apoptotic cells was detected via flow cytometry with the channels set for FITC and PI according to the manufacturer’s instructions. The total apoptosis rate was the sum of early apoptosis, late apoptosis rate, and death rate.

### Potential detection of mitochondrial membrane

The reduction of mitochondrial transmembrane potential is considered an early hallmark of apoptosis. Ferroptosis is also accompanied by the dissipation of mitochondrial membrane potential. Referring to the above 24-well plate transfection condition (20, 40, 60 µg/mL of FeS_2_-based NPs), the transfected cells were irradiated with or without 1064 nm laser (1 W/cm^2^) for 10 min. After 24 h culture, cells digested by trypsin were washed twice with PBS and stained via a Mitochondrial Membrane Potential Assay Kit (Cat. No. C2006, Beyotime, China) with 5,5′,6,6′-tetrachloro-1,1′,3,3′-tetraethylimidacarbocyanine (JC-1) probe for 20 min at 37 °C according to the manufacturer’s instructions. Carbonyl cyanide 3-chlorophenylhydrazone (CCCP) was used as a positive control drug. After washing twice, cells were detected by flow cytometry with the channels set for FITC and PI for mitochondrial membrane potential analysis. Red fluorescence (PI channel) represents a high mitochondrial membrane potential, which depends on the aggregation form of JC-1 in the mitochondria. Green fluorescence (FITC channel) represents a lower potential generated by the monomeric form of JC-1, which appeared in the cytosol after depolarization of the mitochondrial membrane. The percentage of JC-1 monomers in each group was analyzed to measure the loss of mitochondrial membrane potential.

### Cell viability assay

For the assay, cells were plated at a density of 10,000 cells per well in a 96-well plate and allowed to adhere overnight. After transfected with different concentrations of FeS_2_-based NPs (0, 5, 10, 20, 40, 50, 60, 70, 80, 100 µg/mL) for 1 h, cells were treated with or without laser (1064 nm, 1 W/cm^2^) for 10 min. Then, after being cultured for 24 h, cell viability was evaluated with a Cell Counting Kit-8 (CCK-8) (Cat. No. ab228554, Abcam, UK) by measuring the absorbance of 450 nm (*A*_*450*_) under a microplate reader (EPOCH-2, BioTek, USA) according to manufacturer’s instructions. The lower the cell viability, the higher the proliferation inhibition effect. Cell viability was calculated as follows:

Cell viability (%) = *A*_*450*_ of the test well / *A*_*450*_ of the control well (Blk group) × 100%.

### Western blot assay

The treated tumor cells were homogenized and lysed in RIPA buffer (No. P0013B, Beyotime, China) containing 1 mM PMSF and then processed with an ultrasonic probe and subsequent centrifugation to obtain protein extract. Total protein concentration was measured with a BCA assay kit (No. 23,225, Thermo Fisher, USA). Total proteins were separated by SDS-PAGE (12% separation gel and 5% stacking gel) and transferred to polyvinylidene fluoride (PVDF) membranes (90 V × 70 min), followed by blocking with 5% (vol/vol) milk in PBST buffer for 60 min at room temperature. Then the membranes were incubated with primary monoclonal antibodies (1:1000 diluted in 5% milk in PBST buffer) overnight at 4 °C, washed three times with 1 × PBST buffer, and finally incubated with horseradish peroxidase (HRP)-linked secondary antibody (1:3000 diluted in 5% milk in PBST buffer) at room temperature for 90 min. Lastly, the membranes were treated with an ECL kit (Cat. No. 1,863,096 & 1,863,097, Thermo Fisher, USA) and exposed using the FluorChem E ProteinSimple instrument. The expressions of proteins were quantified by Image J. software through gray value analysis with β-actin as an internal control. The primary antibodies were rabbit anti-GPX4 (glutathione peroxidase 4) polyclonal antibody (Cat. No. GB113745, Servicebio, Wuhan, China) and rabbit anti-β-actin monoclonal antibody (Cat. No. 4970, CST, USA). The HRP-linked secondary antibody was goat anti-rabbit IgG polyclonal (Cat. No. 7074, CST, USA).

### In vivo anticancer research

To generate a subcutaneous cell-driven xenograft (CDX) tumor model, MNNG-HOS cells at the exponential growth stage were harvested. Then, five hundred microliters (5 × 10^6^) cells in 100 µL PBS buffer were transplanted subcutaneously into the right flank of five-week-old female athymic Balb/c nu/nu mice (Day 0). When tumor volumes reached the size of approximately 80–100 mm^3^ (Day 3), the mice were randomly divided into six groups of five. Then, for the groups of PBS and FeS_2_@CP, mice received four times intratumoral injections of PBS or FeS_2_@CP NPs (10 mg/kg) on Day 3, 5, 7, and 9, respectively. For the groups of PBS + Laser and FeS_2_@CP + Laser, mice received a single intratumoral injection of PBS or FeS_2_@CP NPs (10 mg/kg) on Day 3. One hour after the injection, the mice were irradiated with a single laser (1064 nm, 1 W/cm^2^, 10 min) on the tumor site. The in vivo photothermal effect of FeS_2_@CP NPs was visualized by the thermal imaging system. The length (*L*: mm) and width (*W*: mm) of tumors and mice body weights were measured every 2 days. The tumor volume (*V*) was calculated as follows: *V* (mm^3^) = 0.5 × *L* × *W*^2^. At the endpoint of therapy, mice were sacrificed by an overdose of isoflurane anesthesia. Then, the tumor masses were stripped from the subcutaneous tissue and weighted for tumor inhibition analysis. Meanwhile, the main organs (heart, liver, spleen, kidney, and lung) of mice were also collected and weighted for organ indices assessment. Finally, histomorphological observations of the collected tumor masses and organs were planned.

### Histology and immunohistochemistry

Ki-67 protein is a nuclear protein related to the cell cycle and an indicator of cell proliferation [26]. The loss of GSH could decrease glutathione peroxidase 4 (GPX4) activity and induce cell ferroptosis [27]. After the treatment experiment, the collected organs and tumors were fixed in a sufficient amount of 4% paraformaldehyde neutral fixative, embedded in paraffin, and cut into 5 μm sections along the maximum cross-section. After dewaxing and hydration, paraffin sections were stained with hematoxylin and eosin (H&E) for histomorphological observation. To assess the proliferation and the ferroptosis of tumor tissue, immunohistochemistry (IHC) was performed on tumor paraffin sections by using Ki-67 antibody (Cat. No. 12,202, CST, USA) and GPX4 antibody (Cat. No. 52,455, CST, USA), respectively. In short, tumor paraffin sections were treated with dewaxing, hydration, antigen repair, endogenous peroxidase inactivation, and non-specific antigen blocking. Then, sections were successively incubated with Ki-67 or GPX4 (glutathione peroxidase 4) primary antibody and biotin-labeled secondary antibody. DAB chromogenic reaction was observed with a DAB Horseradish Peroxidase Color Development Kit (Cat. No. P0202, Beyotime, China) according to the manufacturer’s instructions. Finally, hematoxylin was used for nuclei staining, and observed images were obtained with a Leica inverted microscope. Image-Pro Plus 6.0 software (Media Cybernetics, Inc., Rockville, USA) was used to assess the integrated optical density (IOD) value and the positive area (pixel) of the selected stained region. Mean density = IOD / the positive area.

### TUNEL-IFC assay

Terminal deoxynucleotidyl transferase (TdT)-mediated dUTP nick end labeling immunofluorescence (TUNEL-IFC) assay is commonly used to detect late apoptosis in paraffin sections of tumor tissue. According to the manufacturer’s instructions, a TUNEL Bright Red Apoptosis Detection Kit (Cat. No. A113, Vazyme, China) was used on tumor paraffin sections to evaluate apoptosis in tumor masses. Briefly, after dewaxing and hydration, and treatment with proteinase K, tumor paraffin sections were stained with Bright Red Labeling Mix for TUNEL-IFC staining and then stained with DAPI for nuclear staining. Then, the sections were sealed with anti-fluorescence quenching coverslips. Finally, the stained images of the sections were taken with a Leica fluorescence microscope (Leica M205 FCA, Germany) for TUNEL-IFC analysis. The apoptosis indexes = TUNEL positive cells / total cells × 100%.

### Statistical analysis

Data statistical analyses and graphs collection were generated with GraphPad Prism 7.0 (GraphPad Software Inc., USA). All data were expressed as means ± SD. One-way ANOVA with Tukey’s multiple comparison was used in statistical analyses. A *P* value less than 0.05 was considered statistically significant, and a *P* value otherwise was not statistically significant.

## Results and discussion

### Preparation and characterization of FeS_2_ nanoparticles

**Fig. 1:**
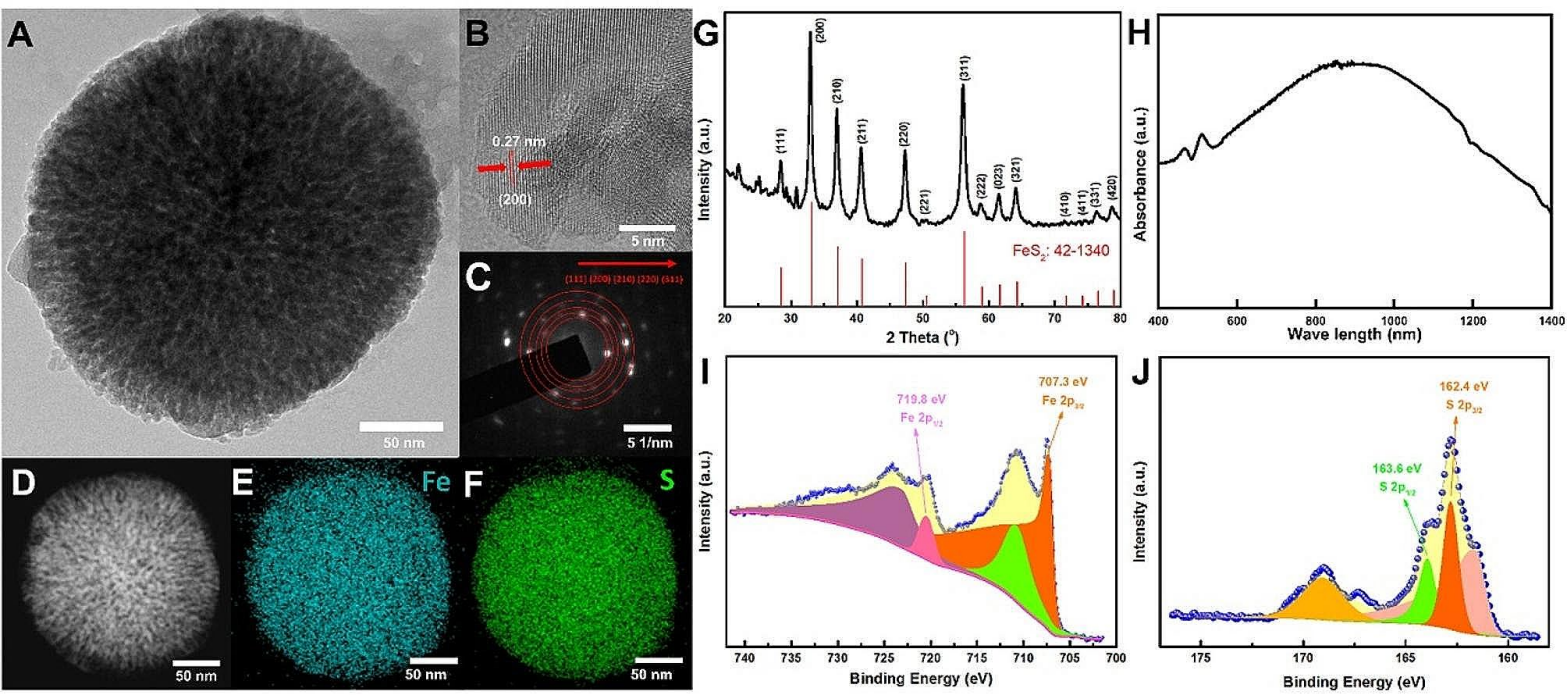
The physico-chemical properties of FeS_2_ NPs. (**A**)The morphology, inner crystal lattice structure and element compositions were observed by TEM. (**B**)The magnified TEM image, (**C**) SAED pattern, and (**D**-**F**) EDX-elemental mapping, scale bar = 50 nm. (**G**) XRD spectrum and (**H**) UV-Vis spectra of FeS_2_ NPs. XPS spectra of FeS_2_ for (**I**) Fe 2p and (**J**) S 2p

To develop sustainable and efficient nano-ferroptosis inducers, pyrite FeS_2_ nanoparticles (NPs) with high Fenton catalytic activity were synthesized via the solvothermal method as Zhang and Meng reported with simple modification [23, 24] (Scheme 1A). The morphology of synthesized FeS_2_ NPs was measured by transmission electron microscopy (TEM) and shown in Fig. 1A. FeS_2_ NPs exhibited a typical spherical shape with a diameter ranging from 75 to 225 nm. The size distribution shown in scanning electron microscopy (SEM) also evidenced the good dispersion and uniform spherical structure with a mean diameter of 175 nm (Fig.S1). The lattice fringes and crystalline structure were also displayed in the high-resolution transmission electron microscope. As shown in Fig. 1B, clear lattice fringes of 0.27 nm can be observed, which correspond to the (211) planes of FeS_2_. The selected area electron diffraction (SAED) confirmed the single-crystalline structure of FeS_2_ (Fig. 1C). The (111), (200), (210), (220), and (311) planes can be recognized obviously. The energy dispersive X-ray spectroscopy (EDS) mapping was also measured to evidence the formation of FeS_2_, as shown in Fig. 1D and F. The homogeneous distribution of iron (Fe) and sulfur (S) confirms the composition of FeS_2_. X-ray diffraction (XRD) spectrum also evidenced the formation of FeS_2_, as shown in Fig. 1G. Typical crystalline peaks for synthesized FeS_2_ corresponded to the cubic structure of pyrite FeS_2_ (PDF: 42-1340). The optical properties of the aqueous dispersion containing FeS_2_ NPs were examined by UV-Vis-NIR spectroscopy. In Fig. 1H, the FeS_2_ exhibited a broad absorption covering the range of 400 to 1400 nm, with a maximum peak at about 900 nm. The valence states were evaluated by X-ray photoelectron spectroscopy (XPS), shown in Fig. 1I and J. The clear signals of Fe 2p and S 2p can be observed in 710.2 eV and 162.7 eV, respectively (Fig. S2). In the Fe 2p spectra, two obvious peaks at 707.3 eV and 719.8 eV can be observed, corresponding to Fe^2+^ of the pyrite FeS_2_. Similarly, in the S 2p spectra, two pronounced peaks located at 162.4 eV and 163.6 eV can be observed, which were assigned to S_2_^2−^ of the pyrite FeS_2_. These results indicated that pyrite FeS_2_ NPs had been successfully synthesized with similar structures to those reported in the literature [23, 24].

### The functional modification of FeS_2_-based NPs

**Fig. 2:**
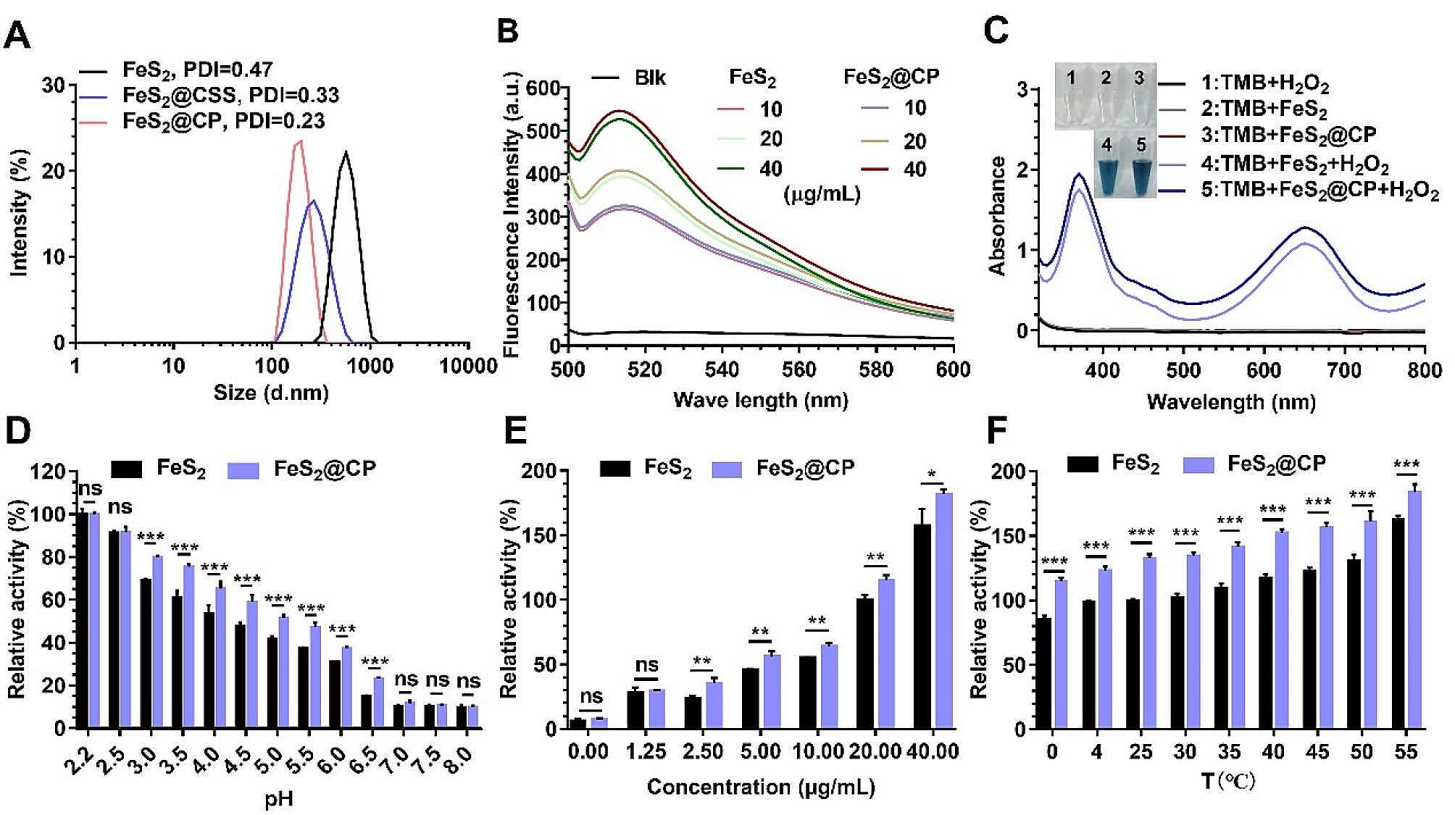
The Catalytic activity assay of FeS_2_-based NPs as Fenton reagents. (**A**) The particle size distributions of FeS_2_-based NPs were detected by DLS. (**B**) Fluorescence intensity of HPF in reaction systems with H_2_O_2_ substrate at different concentrations of FeS_2_ NPs as Fenton catalysts. (**C**) UV-vis absorbance spectra and color changes of TMB in different reaction systems. The relative catalytic activity of FeS_2_-based NPs in reaction systems of (**D**) different pH (**E**) different concentrations, and (F) different temperatures with H_2_O_2_ substrate. All data were expressed as the mean ± SD (*n* = 3. Statistics were done using one-way ANOVA with Tukey multi-comparisons. *p < 0.05, **p < 0.01, ***p < 0.001 and ns, no significance)

Due to the presence of a hydration layer around nanoparticles, the size of hydrodynamic particles measured by dynamic light scattering (DLS) was generally larger than that measured by TEM. However, the average diameter of FeS_2_ NPs obtained by DLS was 796 nm, and the polydispersity index (PDI) was 0.47 (Fig. 2A). The observed abnormal increase in the hydrated particle size suggested a potential for aggregation and instability of FeS_2_ NPs in aqueous environments, which would be detrimental for biological applications. Prior studies have demonstrated that carboxymethyl-dextran (CMD) sodium salt (CSS) modification can reduce the hydrodynamic diameter and polydispersity index (PDI) of iron-based nanoparticles, markedly enhancing their dispersion stability in water [28]. Moreover, modification with PEG-bPEI is known to facilitate the transfection efficiency of nanoparticles in tumor cells due to the positive surface potential giving ability [29]. Hence, we applied CSS and PEG-bPEI modifications to FeS_2_ NPs with the intent to augment their water dispersibility and to enhance transfection efficiency within tumor cells. In addition to Fe and S elements, the gradual addition of C, O, Na, and N elements confirmed that CSS and PEG-bPEI were progressively coated on the surface of FeS_2_ NPs successfully via EDX-elemental overlay mapping analysis (Fig S3). The changes in the surface chemical composition of the nanoparticles obtained at each step are further evidenced the successful preparation of FeS_2_@CSS NPs (FeS_2_ NPs coated with CSS) and FeS_2_@CP NPs (FeS_2_@CSS NPs coated with PEG-bPEI) (Table S1). The continued DLS testing results showed that the mean diameter of FeS_2_@CSS NPs and FeS_2_@CP NPs were reduced to 298 nm and 175 nm, while PDI decreased to 0.33 and 0.23, respectively (Fig. 2A). As shown in Fig. S4A, the zeta potential values (surface potential) of the modified nanoparticles shifted from negative (-21.3 mV of FeS_2_ NPs and − 22.1 mV of FeS_2_@CSS NPs) to positive (+ 20.8 mV of FeS_2_@CP NPs). After overnight standing, no precipitation occurred in the aqueous solution of FeS_2_@CP NPs, while significant precipitation was noted in the FeS_2_ NPs solution (Fig. S4B). These results indicated that the modification could effectively improve the dispersion of FeS_2_-based NPs in an aqueous solution. However, on the one hand, the positive potential on the surface of the modified nanoparticles was expected to be more conducive to the adsorption of the negative potential cell membrane, promoting the uptake and internalization of NPs by tumor cells [30]. On the other hand, positive potential nano-materials tend to accumulate during blood circulation and are isolated by lung, liver and spleen, which is not conducive to the enrichment of NPs in tumor sites [31]. Therefore, intravenous injection may not be the optimal way to administrate the resulting FeS_2_@CP NPs.

### The catalytic activity of FeS_2_-based NPs

Fenton reaction could generate strong cytotoxic •OH to eliminate tumor cells, which have displayed a promising potential in clinical application due to its satisfactory therapeutic effect [32]. The iron-based nanomaterials often show excellent Fenton catalytic activity, which can catalyze the decomposition of •OH from H_2_O_2_ substrate [33]. Hydroxyphenyl fluorescein (HPF) was used to determine the produced·•OH from H_2_O_2_ substrate. As the concentration of FeS_2_ NPs or FeS_2_@CP NPs increased, the gradually enhanced fluorescence intensity from oxidized HPF was observed (Fig. 2B). Meanwhile, the colorimetric method based on the oxidation of 3,3′,5,5′-tetramethylbenzidine (TMB) was employed to examine the capacity of FeS_2_-based NPs to transform H_2_O_2_ into •OH. As shown in Fig. 2C, FeS_2_ NPs, FeS_2_@CP NPs, and H_2_O_2_ alone could not oxidize TMB into oxidized TMB (oxTMB, blue color). However, in the presence of FeS_2_-based NPs and H_2_O_2_, a bright blue color was observed with a typical bimodal UV-visible spectrum at 370 and 652 nm. These results together displayed that FeS_2_ NPs and FeS_2_@CP NPs could act as Fenton reagents to catalyze the production of •OH from the H_2_O_2_ substrate (Fig. 2B and C). The influence of pH value on the catalytic activity of FeS_2_ NPs was analyzed in Na_2_HPO_4_-citrate buffer (Fig. S5A). The results indicated that the catalytic activity of FeS_2_ NPs increased with the decrease in pH value. Furthermore, FeS_2_ NPs retained strong activity in pH 4.0–5.0 buffer but almost no activity in buffer with pH less than 7. This basis indicated that the FeS_2_ NPs were most active when they localized in endo-lysosomes with an acidic environment (pH 4.5-5.0) but were inactive in normal body fluid (∼ pH 7.4) [9]. Meanwhile, FeS_2_ NPs exhibited strong catalytic activity in a concentration-dependent manner (Fig. S5B). More importantly, the catalytic activity of FeS_2_ NPs increased with the reaction temperature (Fig. S5C). This temperature-dependent activity suggested that raising the temperature could accelerate the catalytic decomposition of H_2_O_2_ by FeS_2_ NPs to produce more •OH.

Next, the catalytic activities of FeS_2_-based NPs in different pH buffers were evaluated using FeS_2_ NPs activity in pH 2.2 buffer as the highest activity. The result showed that the modified FeS_2_@CP NPs had higher catalytic activity than FeS_2_ NPs in the weak or moderately strong acidic buffer. No matter whether modified or not, FeS_2_ NPs and FeS_2_@CP NPs had weak activity in a neutral or alkaline environment (Fig. 2D). Accordingly, compared with FeS_2_ NPs, the relative catalytic activity of FeS_2_@CP NPs significantly increased under the same concentration of NPs and the same reaction temperature (Fig. 2E and F). The reaction of FeS_2_ NPs at a concentration of 20 µg/mL in pH 4.5 buffer for 10 min at room temperature (RT) yielded 100% activity (Fig. 2E and F). These results indicated that the Fenton catalytic activities of modified FeS_2_@CP NPs were significantly enhanced. This might be due to the enhanced dispersion after CSS and PEG-bPEI modification since the activities of catalysts are related to their active surface areas in contact with the substrates [34].

### Photothermal effect of FeS_2_@CP NPs

**Fig. 3:**
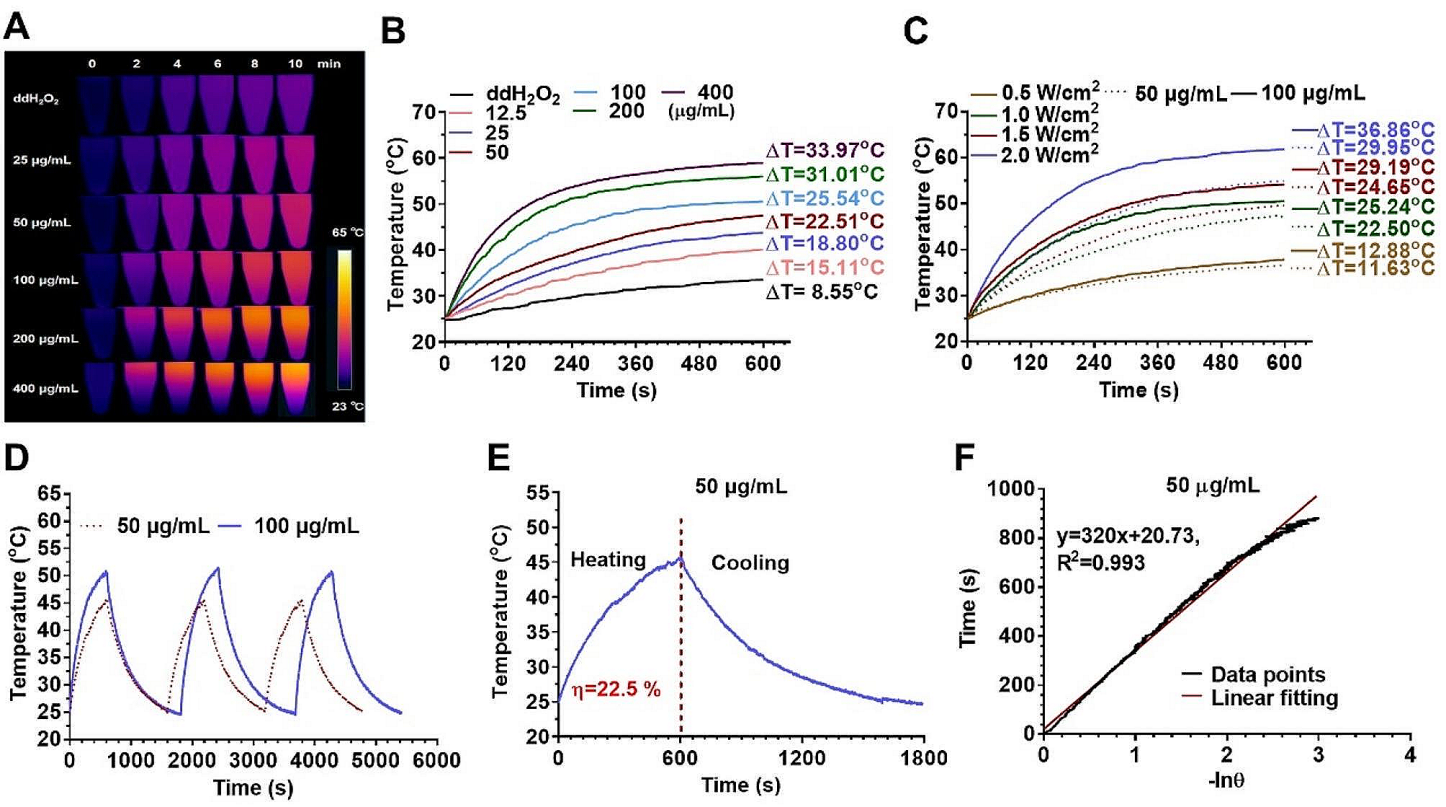
The photothermal response of FeS_2_@CP NPs. (**A**) The temperature gradience and (**B**) the temperature elevation curve of FeS_2_@CP NPs in different concentrations under laser irradiation (1064 nm, 1 W/cm^2^) for 10 min. (**C**) The temperature elevation curve of FeS_2_@CP NPs under laser irradiation with different power densities for 10 min. (**D**) The Photostability of FeS_2_@CP NPs (50 µg/mL and 100 µg/mL) was evaluated by performing the lasering and cooling cycle several times. (**E**) The heating curve of FeS_2_@CP NPs (50 µg/mL) under laser irradiation and natural cooling process after laser shutdown. (**F**) Linear time data versus -ln(θ) obtained from the cooling period of the panel (**E**) to calculate the PCE (η, presented in panel (**E**)) of FeS_2_@CP NPs

Metal-based nanomaterials, such as iron-based nanomaterials, often exhibit excellent photothermal conversion properties [35]. Before and after modification, all the absorption peaks of FeS_2_-based NPs ranged from 400 to 1400 nm (Fig. S6), which indicated that FeS_2_-based NPs had photothermal conversion properties both under NIR-I region and NIR-II region lasers. Because the NIR-II laser has a greater advantage in clinical PTT due to higher MPE and deeper tissue penetration [36, 37], the photothermal responses of FeS_2_@CP NPs were evaluated under the irradiation of a 1064 nm laser. With a laser power density of 1 W/cm^2^, and the FeS_2_ concentration varied from 12.5 to 400 µg/mL, the temperature of the aqueous dispersions increased by 15.11 ℃ to 33.97 ℃ within 10 min. However, the water temperature only increased by 8.55 ℃ under the same condition (Fig. 3A and B). When changing the laser power density from 0.5 to 2.0 W/cm^2^, FeS_2_@CP aqueous solution at concentrations of 50 µg/mL and 100 µg/mL exhibited temperature increments from 11.63 to 29.95 ℃ and 12.88 to 36.86 ℃, respectively (Fig. 3C). These results revealed that the temperature elevation of FeS_2_@CP had a positive correlation with the concentration of samples and the irradiation power (Fig. 3A and C). In addition, the two concentrations of FeS_2_@CP solution also showed good photothermal stability by repeating the lasering and cooling cycles three times (Fig. 3D). The PCE (η) is a key parameter to measure the light-to-heat conversion sensitivity of photothermal materials. To quantify this parameter of FeS_2_@CP, the heating curve of FeS_2_@CP solution (50 µg /mL) under laser irradiation and natural cooling process after laser shutdown was performed on (Fig. 3E) and linear time data versus -ln(θ) were obtained (Fig. 3F). Finally, η was calculated to be 22.5% (presented in Fig. 3E) according to Eqs. 1–4 in the experimental section. The above data indicated that high PCE and good photothermal stability made FeS_2_@CP NPs a broad application prospect in PTT.

### NIR laser light-enhanced catalytic activity of FeS_2_-based NPs

**Fig. 4:**
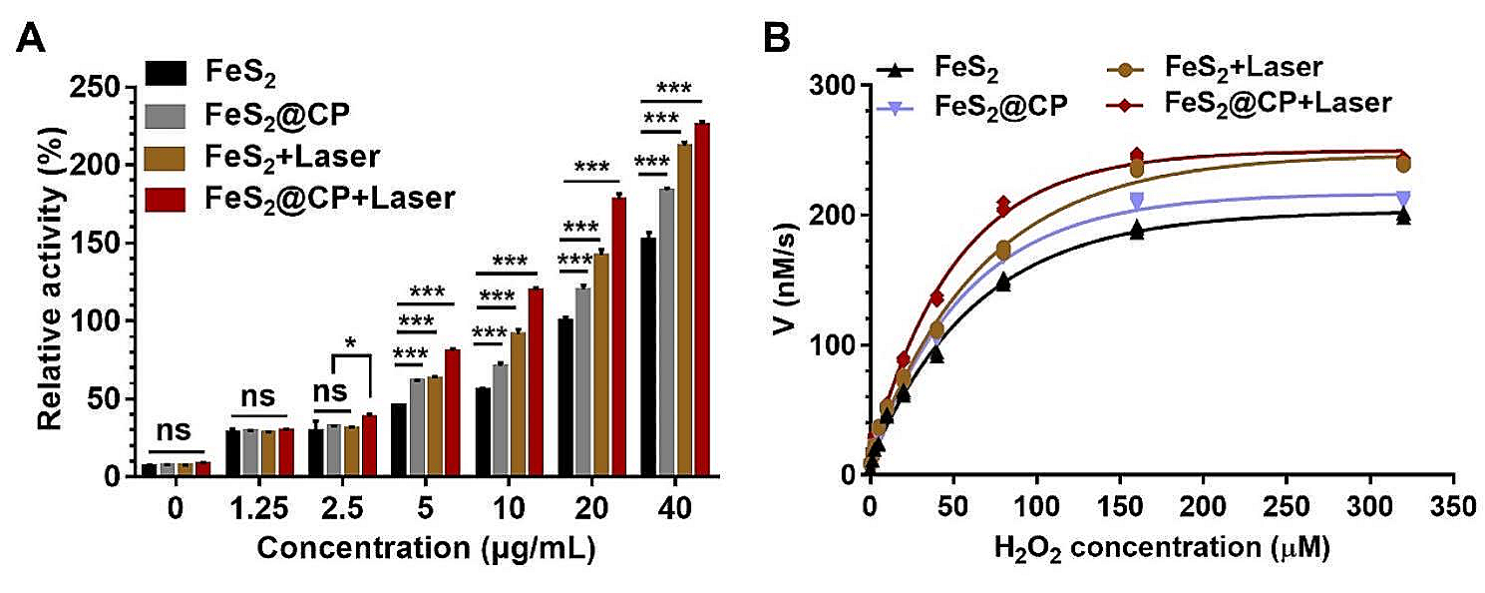
The catalytic activity and a kinetic assay of FeS_2_-based NPs with or without laser irradiation. (**A**) Enhanced concentration-dependent and relative catalytic activity of FeS_2_-based NPs on H_2_O_2_ substrate by laser irradiation. (L) The kinetic assay of FeS_2_-based NPs on H_2_O_2_ substrate with or without laser irradiation

The above results robustly demonstrated that FeS_2_-based NPs could be used as Fenton catalytic reagents and photothermal reagents. Then, we quantified the catalytic activity of FeS_2_-based NPs with or without a 1064 nm laser. As demonstrated in Fig. 4A and B, the catalytic activity of FeS_2_ NPs and FeS_2_@CP NPs both were significantly enhanced with NIR-II irradiation, which suggested that the heat from the photothermal effect could obviously boost the generation of •OH. In addition, by using Eqs. 5–7 in the experimental section, the enzyme kinetics analysis of FeS_2_-based NPs with H_2_O_2_ as substrate was carried out in pH 4.5 buffer with 20 µg/mL of NPs at 37 ℃ with or without laser. After the reaction for 10 min, FeS_2_-based NPs displayed typical Michaelis-Menten curves of enzyme kinetics drawn within H_2_O_2_ concentrations. The lower the Michaelis constant (*K*_*M*_), the higher the substrate affinity of the catalyst. The higher the value of maximal reaction velocity (*V*_*max*_) and catalytic constant (*k*_*cat*_), the faster the catalytic rate. Finally, *k*_*cat*_/*K*_*M*_ reflects the catalytic efficiency of the catalyst. Without NIR-II irradiation, *V*_*max*_, *K*_*M*_, *k*_*cat*,_ and *k*_*cat*_/*K*_*M*_ of FeS_2_ NPs were 202.9 nM/s, 46.51 µM, 8.57 × 10^4^/s, and 1.84 × 10^9^, respectively. While these values of modified FeS_2_@CP NPs were improved to 216.6 nM/s, 40.06 µM, 9.15 × 10^4^/s, and 2.28 × 10^9^, respectively (Table 1). These values of FeS_2_-based NPs were close to that of high-performance pyrite nanozymes reported by Meng, which showed thousands of times higher catalytic efficiency than classical Fe_3_O_4_ nanozymes and natural horseradish peroxidase (HRP) [23]. The high substrate affinity and catalytic efficiency of FeS_2_-based NPs meant that they could rapidly initiate the Fenton reaction in the TME of 10–100 µM of H_2_O_2_ to produce large amounts of •OH to kill tumor cells. This is the key to the success of CDT based on the Fenton catalyst. Importantly, NIR-II irradiation would further improve these values of the two NPs to 246.8 nM/s, 44.16 µM, 10.42 × 10^4^/s, 2.36 × 10^9^, and 249.6 nM/s, 35.15 µM, 10.52 × 10^4^/s and 2.99 × 10^9^, respectively. The results confirmed that NIR II irradiation could not only significantly improve the substrate affinity of FeS_2_-based NPs but also increase the catalytic efficiency of FeS_2_-based NPs by nearly 1.3 times to produce more •OH. This provided a basis for the application of these nano-Fenton catalysts in photothermal ablation and NIR II-enhanced chemodynamic synergetic therapy.

**Table 1:** The kinetic parameters of FeS_2_-based NPs on H_2_O_2_ substrate with or without laser irradiation

	V_max_ (nM/s)	K_M_ (µM)	k_cat_ (s^− 1^)	k_cat_/K_M_ (s^− 1^M^− 1^)
**FeS**_**2**_	202.9	46.51	8.57 × 10^4^	1.84 × 10^9^
**FeS**_**2**_ **@CP**	216.6	40.06	9.15 × 10^4^	2.28 × 10^9^
**FeS**_**2**_ **+ Laser**	246.8	44.16	10.42 × 10^4^	2.36 × 10^9^
**FeS**_**2**_ **@CP + Laser**	249.6	35.15	10.52 × 10^4^	2.99 × 10^9^

### Cell uptake and intracellular distribution of FeS_2_-based NPs

**Fig. 5:**
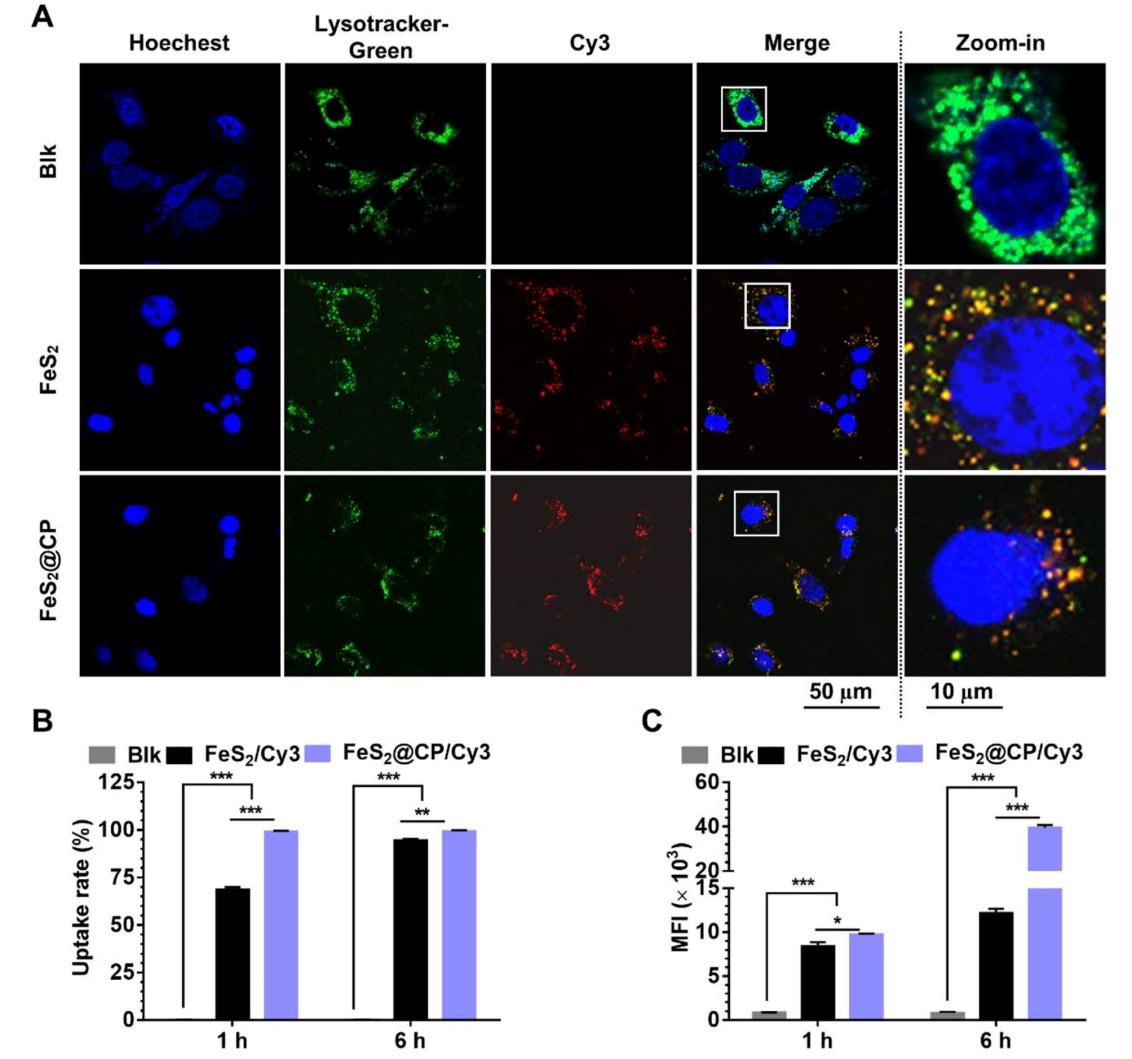
Intracellular uptake and subcellular localization analysis of FeS_2_-based NPs. (**A**) Subcellular localization of FeS_2_-based NPs on MNNG-HOS cells at 1 h post-transfection. Blue: cell nuclei were stained by Hoechst 33,342; Green: Endosomes or lysosomes stained by Lyso-Tracker Green; Red: NPs labeled with Cy3, Merge: Hoechst + Lyso-Tracker Green + Cy3, scale bar = 50/10 µm. (**B**) Quantitative analysis of cell uptake rates and (**C**) the mean fluorescence intensity (MFI) analysis in MNNG-HOS cells treated with FeS_2_-based NPs detected by flow cytometry. Blk: Blank. All data were expressed as the mean ± SD (*n* = 3. Statistics were done using one-way ANOVA with Tukey multi-comparisons. *p < 0.05, **p < 0.01, ***p < 0.001 and ns, no significance)

The intracellular uptake and subcellular localization experiments performed on osteosarcoma cells were observed by confocal laser scanning microscope (CLSM) and flow cytometry. Results of endo-lysosome and nanoparticle colocalization experiments of MNNG-HOS cells displayed that endo-lysosomes and FeS_2_-based NPs were highly colocalized (Fig. 5A). The result showed that FeS_2_-based NPs entered into cells via the endocytosis pathway. Moreover, with the extension of transfection time, the uptake rate of Cy3-labeled nanoparticles by osteosarcoma cells gradually increased, and the Mean fluorescence intensity (MFI) of Cy3 increased at the same time (Fig. 5B and C and Fig. S6). For MNNG-HOS cells treated with FeS_2_ NPs, the average uptake rate was 67.71% at 1 h post-transfection, which increased to 94.56% at 6 h post-transfection. While the uptake rates of cells treated with FeS_2_@CP NPs were 99.16% and 99.36% at 1 and 6 h post-transfection, respectively (Fig. 5B), which were significantly higher than those of cells treated with FeS_2_ NPs. Meanwhile, from 1 to 6 h after transfection, the MFI of Cy3 in cells of group FeS_2_ increased from 8.40 × 10^3^ to 12.15 × 10^3^, while it increased from 9.75 × 10^3^ to 39.61 × 10^3^ in group FeS_2_@CP (Fig. 5C). In addition, both the uptake rate and the Cy3 MFI of U2-OS cells in the FeS_2_@CP group were significantly higher than those of cells in the FeS_2_ group (Fig. S7). These results showed that osteosarcoma cells could efficiently take up FeS_2_ NPs and FeS_2_@CP NPs through the endocytosis pathway, and the latter had higher uptake efficiency. Importantly, the optimal environment for the Fenton reaction is pH 2.0 ∼ 4.5 [32]. The endosomes would subsequently carry the FeS_2_-based NPs into the acid lysosomal compartments, where the acidic environment (pH 4.5-5.0) was suitable for the Fenton catalyst activity of FeS_2_-based NPs.

### Biosafety analysis of FeS_2_-based NPs

Good biocompatibility and low toxicity are the precursors for the clinical application of nano-reagents. In general, the neutral (∼ pH 7.4) and low H_2_O_2_ environment of normal tissues inhibit the Fenton reaction, while the acidity and overproduce of H_2_O_2_ in TEM favor the Fenton reaction. This results in the generation of •OH only in tumor tissues while ensuring the safety of normal tissues [38]. Biosafety evaluation was carried out before examining the antitumor activity of FeS_2_-based NPs both in vitro and in vivo. As shown in Fig. S8A, the FeS_2_ NPs and FeS_2_@CP NPs were degraded gradually both in pH 4.5 and pH 7.4 buffers, and the degradation was faster in the pH 4.5 buffer. This indicated that the FeS_2_-based NPs were biodegradable in both lysosomal acidic and neutral physiological environments, and the former environment was more conducive to degradation. The hemolysis experiment showed that the hemolysis rates of the FeS_2_-based NPs were very low at a concentration of fewer than 200 µg/mL (Fig. S8B). Crucially, our findings indicate that the FeS_2_-based nanoparticles were effective in selectively targeting osteosarcoma cell lines MNNG-HOS and U2-OS, while demonstrating negligible cytotoxicity towards the normal HUVEC cell line (Fig. S8C). The biodegradability, minimal hemolysis rates, and low toxicity towards normal cells collectively suggest a promising biocompatibility profile for the FeS_2_-based nanoparticles.

Then, the in vivo biosafety assessments were performed in healthy Balb/c mice administrated intravenously with 60 mg/kg of the FeS_2_-based NPs. During a 15-day monitoring period, there was no abnormality in diet, water drinking, and physical behavior of all the treated mice from different groups. Compared to the mice treated with PBS, mice treated with either FeS_2_ NPs or FeS_2_@CP NPs illustrated no significant difference in blood cell counts (19 blood routine values in Fig. S9) and serum biochemistry (16 blood biochemical indexes in Fig. S10) at the end of monitoring. The results indicated that FeS_2_-based NPs treatment did not induce inflammatory responses in vivo and had no significant hematotoxicity and hepatotoxicity. Meanwhile, there were no significant changes in the body weight of mice in each group during the monitoring (Fig. S11A), and no significant difference in major organ indexes (heart, liver, spleen, kidney, and lung) at the end of monitoring (Fig. S11B). Furthermore, histology results from H&E staining of the above organs’ paraffine sections exhibited that either FeS_2_ NPs or FeS_2_@CP NPs had no significant toxic and side effects on the major organs of mice (Fig. S12). Such results indicated that FeS_2_-based NPs were biocompatible and well-tolerated in healthy mice, which enables them to have great potential for clinical application.

### In vitro anticancer effect

**Fig. 6:**
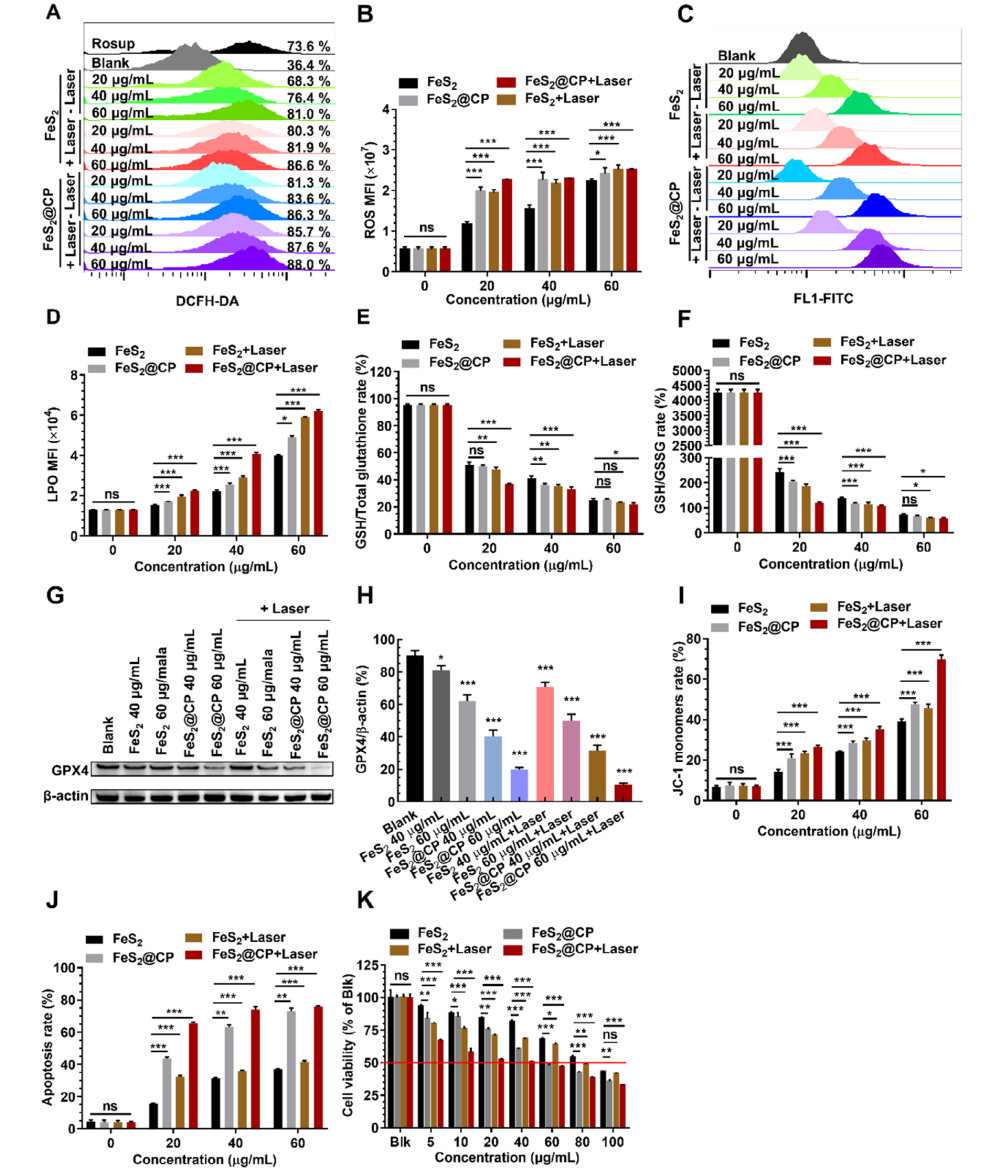
Anticancer efficacy of FeS_2_-based NPs in MNNG-HOS cells. Intracellular ROS level assessed with (**A**) the percentages of cells producing ROS and (**B**) the MFI of ROS detected by flow cytometry with DCFH-DA probe in MNNG-HOS cells after FeS_2_-based NPs treatment for 24 h. (**C**) Intracellular LPO level detected by flow cytometry with C11-BODIPY 581/591 probe in MNNG-HOS cells after FeS_2_-based NPs treatment for 24 h. (**D**) Quantitative MFI analysis of LPO recorded in panel (**C**). Loss of intracellular GSH assessed with (**E**) the rate of GSH/total glutathione and (**F**) the rate of GSH/GSSG in MNNG-HOS cells after FeS_2_-based NPs treatment for 24 h. (**G**) Western blot analysis of GPX4 expression in MNNG-HOS cells treated with different nano-formulations for 24 h with or without laser irradiation, and (H) GPX4 protein expression was quantified and normalized with β-actin. (**I**) Quantitative analysis of JC-1 monomers rate to assess the mitochondrial membrane potential of MNNG-HOS cells induced by FeS_2_-based NPs treatment for 24 h. (**J**) Quantitative analysis of MNNG-HOS cells apoptosis induced by FeS_2_-based NPs treatment for 24 h. (**K**) Cell viability of MNNG-HOS cells was analyzed by a CCK-8 assay after the treatment with FeS_2_-based NPs for 24 h. All data were expressed as the mean ± SD (*n* = 3. Statistics were done using one-way ANOVA with Tukey multi-comparisons. *p < 0.05, **p < 0.01, ***p < 0.001 and ns, no significance)

Tumor cells will continue to produce and accumulate H_2_O_2,_ resulting in a higher concentration in TEM [39]. Thus, Fenton catalytic-based tumor CDT strategies have been developed by catalyzing the endogenous H_2_O_2_ in tumor cells to high toxic •OH [23, 24, 33]. However, the amount of •OH generated from the Fenton reaction limited the therapeutic effects. FeS_2_-based NPs displayed excellent biosafety, high PCE, and high efficiency & NIR II-enhanced Fenton catalytic activity, which enabled FeS_2_-based NPs to combine PTT and CDT. So, the application of FeS_2_-based NPs in photothermal ablation and nano-catalytic synergetic therapy was studied on osteosarcoma cells of MNNG-HOS and U2-OS. The production of •OH from endogenous H_2_O_2_ substrate catalyzed by Fenton catalyst can induce the increase of intracellular ROS level, which can be detected by 2′,7′-dichlorofluorescein diacetate (DCFH-DA). After 24 h of FeS_2_-based NPs treatment, all the percentages of cells producing ROS and the MFI of ROS in osteosarcoma cells (MNNG-HOS cells and U2-OS cells) increased in a concentration-dependent manner. The modification of FeS_2_ NPs to FeS_2_@CP NPs would promote the generation of ROS at each concentration. NIR irradiation would further promote the generation of ROS at low concentrations of NPs, but the promotion was not obvious at high concentrations of NPs (Fig. 6A and B and Fig. S13A-S13B). The production of lipid radicals or •OH can initiate nonenzymatic lipid peroxidation (LPO), which may be driven by the Fenton reaction with iron as a catalyst. Meanwhile, LPO levels in osteosarcoma cells also increased with the increase of FeS_2_-based NPs concentration, and the modification of NPs and the combination of irradiation would promote the production of LPO (Fig. 6C and D and Fig. S13C-S13D). Due to the efficient activity of the Fenton catalyst in ROS generation, intracellular GSH depletion may occur [18, 19]. As expected, both the GSH/total glutathione rate and the glutathione/oxidized glutathione (GSH/GSSG) rate in FeS_2_@CP NPs-treated cells were significantly lower than that in FeS_2_ NPs-treated cells, and NIR irradiation promotes the GSH loss at each concentration of NPs (Fig. 6E and F and Fig. S13E-S13F). The entry of Fe^2+^ into cells initiates the Fenton reaction, which increases intracellular ROS level, and LPO, which is one of the main mechanisms of ferroptosis. Intracellular GSH depletion may also lead to decreased glutathione peroxidase 4 (GPX4) activity and ferroptosis. Corresponding Western blot results indicated that GPX4 expression in osteosarcoma cells reduced with the increase of FeS_2_-based NPs concentration, and the modification and the combination of irradiation could further reduce the expression of GPX4 (Fig. 6G and H and Fig. S13G-S13H). The results above together proven that the modified FeS_2_@CP NPs, as Fenton catalysts, could more efficaciously kill the tumor cells by catalyzing H_2_O_2_ to produce toxic •OH and deplete GSH to initiate ferroptosis mechanism, and NIR-II irradiation would further promote this process.

In addition, a high concentration of ROS can activate external or intrinsic apoptotic pathways to induce apoptosis [40]. After 24 h treatment with FeS_2_-based NPs, the ratios of 5,5′,6,6′-tetrachloro-1,1′,3,3′-tetraethylimidacarbocyanine (JC-1) monomers in osteosarcoma cells increased in a concentration-dependent manner. The modification of FeS_2_ NPs to FeS_2_@CP NPs and NIR II irradiation would promote the increase of JC-1 at each concentration (Fig. 6I & Fig.S13 and Fig. S13I & Fig. S15). The increase of JC-1 monomers means the decrease of mitochondrial membrane potential, which is a landmark early event in apoptosis. As expected, the apoptosis rates of cells induced by FeS_2_-based NPs treatment also increased with the increase of NPs concentration, and the modification of NPs and the combination of NIR irradiation further promoted apoptosis (Fig. 6J & Fig.S16 and Fig. S13J & Fig. S17). The results indicated that both cell apoptosis and ferroptosis were involved in the cell death mechanism caused by FeS_2_-based NPs treatment, and NIR II irradiation could promote the progression of cell death. This dual death mechanism would hopefully overcome the problem of tumor cells escaping drug killing through anti-apoptosis or anti-ferroptosis death. Finally, FeS_2_-based NPs treatments combined with photothermal ablation and nano-catalytic therapy could strongly inhibit tumor cell proliferation (Fig. 6K and Fig. S13K). All the results above indicated that modified FeS_2_@CP NPs could effectively drive apoptosis and ferroptosis in photothermal ablation/NIR II-enhanced nano-catalytic synergistic therapy for killing osteosarcoma cells.

### In vivo therapeutic effect and pathological analysis

The results above showed that compared with FeS_2_ NPs, the modified FeS_2_@CP NPs were superior in dispersion, Fenton catalytic activity, and in vitro antitumor activity. Accordingly, FeS_2_@CP NPs were selected to carry out an in vivo antitumor therapy trial. To evaluate the antitumor effect of FeS_2_@CP NPs in vivo, an MNNG-HOS cell line drive xenograft (CDX) subcutaneous tumor model was established in Balb/c nude mice. Before the treatment trial, the photothermal images of tumor-bearing mice intratumorally injected with PBS, FeS_2_@CP NPs (10 mg/kg) were recorded via an infrared thermal imaging camera with 1 W/cm^2^ 1064 nm laser (Fig.S18A). As seen from Fig. S18B, the temperature in the tumor area of the FeS_2_@CP NPs-treated group increased rapidly to over 55 ℃ in 10 min. However, the temperature in the tumor area of the mice injected with PBS, the control group, only had a slight increment under the same condition.

**Fig. 7:**
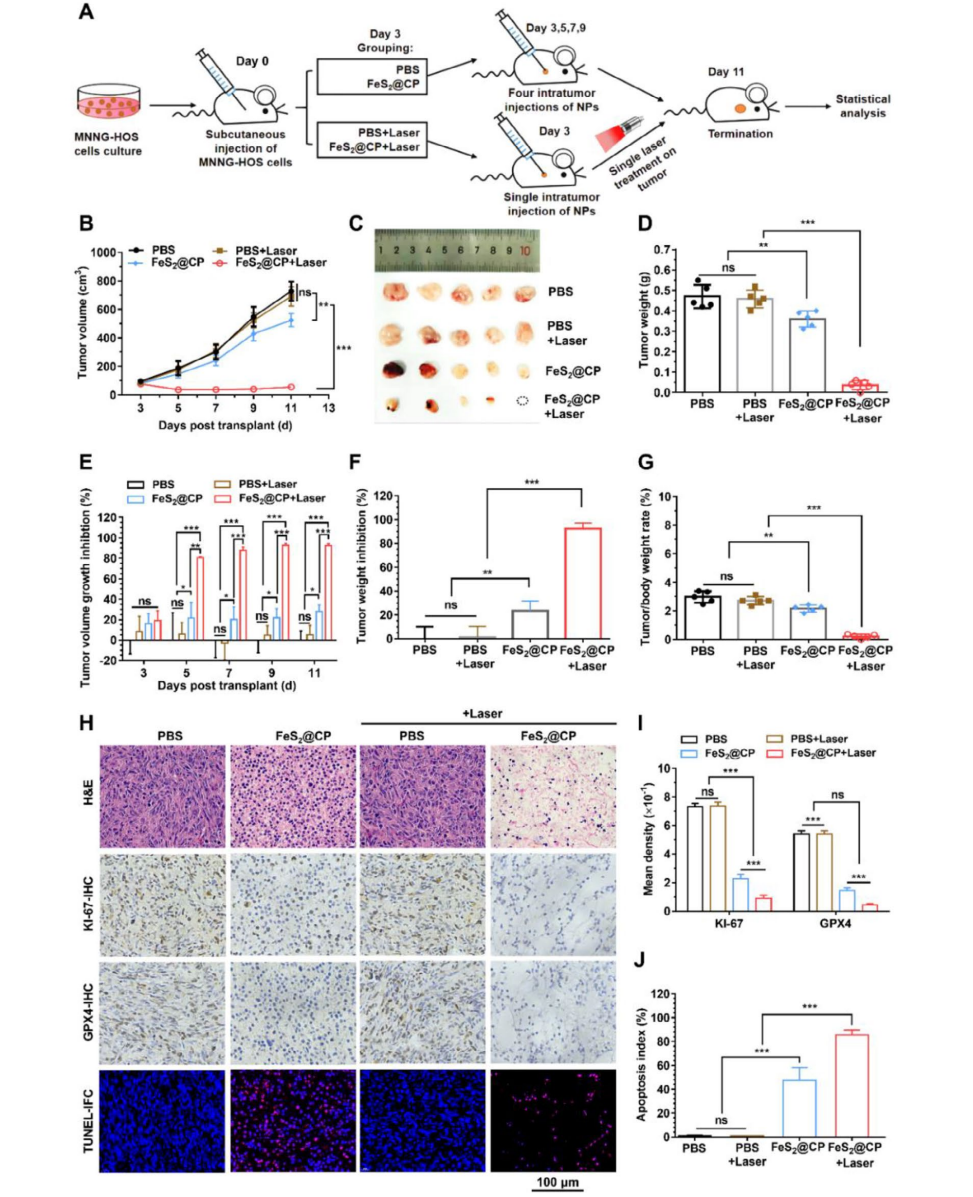
In vivo antitumor efficacy of FeS_2_-based NPs in an MNNG-HOS CDX model. (**A**) Treatment schedule and grouping information on the antitumor study performed on an MNNG-HOS CDX model. (**B**) Tumor growth curves were recorded on tumor-bearing mice during the treatment trial. (**C**) Size and (**D**) weight of isolated tumors from mice in the tested groups at the end time point. (**E**) The tumor volume growth inhibition rate of mice treated with different NPs. (**F**) The tumor weight inhibition and (**G**) the tumor/body weight ratio in each test group at the treatment endpoint. (**H**) Representative microphotographs of H&E staining, Ki-67-immune-histochemical (Ki-67 IHC) staining, GXP4-immune-histochemical (GXP4 IHC) staining and TUNEL-immunofluorescence (TUNEL-IFC) staining of the tumor sections collected from the treated mice at the endpoint, scale bar = 100 μm. (I) Analysis of the mean density of Ki-67-IHC and GXP4-IHC. (J) Analysis of the apoptosis index of TUNEL-IFC. All data were expressed as the mean ± SD (*n* = 5, one-way ANOVA with Tukey multi-comparisons. *p < 0.05, **p < 0.01, ***p < 0.001 and ns, no significance)

Then, according to the therapeutic assessment schedule in Fig. 7A, the tumor-bearing mice were treated with the above dose of FeS_2_@CP NPs administrated by intratumoral injection. During the treatment, the mice in groups of PBS and FeS_2_@CP received one injection every 2 days for a total of 4 times. While the mice in groups of PBS + Laser and FeS_2_@CP + Laser received a single injection and a single 10 min irradiation on the tumor site. As shown in Fig. 7B and G, four treatments with FeS_2_@CP NPs only significantly inhibited the MNNG-HOS-tumor growth. However, combined with NIR II-laser irradiation, only once treatment with FeS_2_@CP NPs led to a dramatic effect on tumor ablation, to the extent that tumors barely grew. Accordingly, FeS_2_@CP NPs administration showed a significant tumor volume inhibition, and irradiation greatly enhanced such inhibition effect. After combined laser irradiation, the maximum inhibition rate of the FeS_2_@CP + Laser group was as high as 92.44% during treatment (Fig. 7E). At the end of the treatment, the final average tumor weights were 0.470 g, 0.456 g, 0.361 g and 0.035 g in the groups of PBS, PBS + Laser, FeS_2_@CP and FeS_2_@CP + Laser, respectively (Fig. 7D). The corresponding average relative tumor weight inhibition rates in the four groups were 0.004%, 1.403%, 23.41%, and 92.34%, respectively, at the treatment endpoint (Fig. 7F). Compared with PBS or PBS + irradiation, administration with FeS_2_@CP NPs significantly reduced the tumor/body weight rate of mice, and combination with NIR-irradiation further enhanced the reduction (Fig. 7G). As seen in Fig. 7H and I, H&E staining displayed that the tumor tissues in the FeS_2_@CP-treated group displayed large areas of necrosis, nucleus shrinkage, incomplete cell membranes, and loss of normal morphology. Meanwhile, negative staining results of Ki-67-IHC and GXP4-IHC with positive results of TUNEL-IFC staining were shown in the corresponding necrotic areas. Moreover, the combination of NIR-laser further promoted nucleus fragmentation, cell disintegration, and tissue loss (Fig. 7H), followed by the lower mean density of KI-67-IHC and GXP4-IHC, and higher apoptosis indexes of TUNEL-IFC (Fig. 7I). However, there was no significant difference in the above four tumor staining results of the groups treated with PBS only and PBS + Laser (Fig. 7H and I). These results were consistent with the results of in vitro antitumor experiments. FeS_2_@CP NPs treatment combined with photothermal therapy induced both cell apoptosis and ferroptosis. And the dual mechanism of cell death was mediated by both LPO due to large amounts of •OH production and GPX4 inactivation due to GSH depletion, which effectively inhibited the growth of osteosarcoma in vitro and in vivo (Fig. 6I & Fig. S12I and Fig. 7B and G). Importantly, the combination therapy with FeS_2_@CP and NIR-laser exhibited excellent tumor suppression effects in MNNG-HOS-bearing mice, but it did not cause abnormalities in the daily behavior, body weight (Fig. S19A), main organ index (Fig. S19B) or main organ histomorphology (Fig. S20) of mice. Taken together, FeS_2_@CP NPs exhibited excellent photothermal ablation and NIR II-enhanced nano-catalytic synergistic antitumor activity in vivo without toxic and side effects.

## Conclusion

In summary, a bi-functional therapeutic system has been constructed by using FeS_2_@CP NPs as synergetic therapeutic catalysts for both PTT and NIR II-enhanced CDT against osteosarcoma. The superior catalytic performance in catalyzing limited H_2_O_2_ in TME to •OH in Fenton reaction with *V*_*max*_, *K*_*M*,_ and *K*_*cat*_*/K*_*M*_ of 216.6 nM/s, 40.06 μm, and 2.28 × 10^9^ s^− 1^M^− 1^ could be ascribed to surface modification on FeS_2_@CP NPs. Thus, FeS_2_@CP-based CDT efficaciously killed the tumor cells by initiating a dual cell death mechanism of apoptosis and ferroptosis, which was mediated by both LPO due to large amounts of •OH production and GPX4 inactivation due to GSH depletion. Furthermore, due to the high photothermal-conversion efficiency under the 1064 nm laser irradiation, heat generated by FeS_2_@CP-based PTT could not only ablate tumors directly but also enhance •OH production and GSH depletion for CDT. Finally, the combination of FeS_2_@CP NPs-based PTT and CDT strengthened the dual mechanism of cell death and effectively inhibited the growth of osteosarcoma in vitro and in vivo. Besides, the high biocompatibility of FeS_2_@CP NPs was also confirmed by both in vitro and in vivo studies. This work highlighted the great potential of the synergistic effect of PTT combined with NIR II-enhanced CDT, providing a new concept of designing high-performance nano-catalysts for effective cancer treatment.

### Electronic supplementary material

Below is the link to the electronic supplementary material.

Supplementary Material 1

## Data Availability

The data are all available upon a reasonable request.
