# Supplementary material for: High-performance pyrite nano-catalyst driven photothermal/chemodynamic synergistic therapy for Osteosarcoma
Source: J Nanobiotechnology. 2024 Apr 1;22:141. doi: 10.1186/s12951-024-02419-2 (PMC10983657; doi:10.1186/s12951-024-02419-2)
Supplement: Supplementary file 1 — Supplementary Material 1 [file 12951_2024_2419_MOESM1_ESM.docx]

**SUPPLEMENTARY MATERIALS**

**High-performance pyrite nano-catalyst driven photothermal/chemodynamic synergistic therapy for Osteosarcoma**

Meirong Li^1,2,#^, Minghua Wang^3,#^, Junfeng Huang^4,#^, Shiqi Tang^2^, Jingyu Yang^2^, Zhourui Xu^2^, Gaixia Xu^2^, Xin Chen^2^, Jia Liu^1,*^, Chengbin Yang^2,*^

*Correspondence: [liujia870702@126.com](mailto:liujia870702@126.com); [cbyang@szu.edu.cn](mailto:cbyang@szu.edu.cn)

^1^Central Laboratory, The Second Affiliated Hospital of the Chinese University of Hong Kong, Shenzhen & Longgang District People’s Hospital of Shenzhen, Shenzhen 518172, Guangdong, P. R. China

^2^Guangdong Key Laboratory for Biomedical Measurements and Ultrasound Imaging, School of Biomedical Engineering, Shenzhen University Medical School, Shenzhen University, Shenzhen 518060, Guangdong, P. R. China

^3^Pathology department, The Second Affiliated Hospital of the Chinese University of Hong Kong, Shenzhen & Longgang District People’s Hospital of Shenzhen, Shenzhen 518172, Guangdong, P. R. China

^4^School of Mechanical Engineering, Dongguan University of Technology, Dongguan 523808, Guangdong, P. R. China.

**
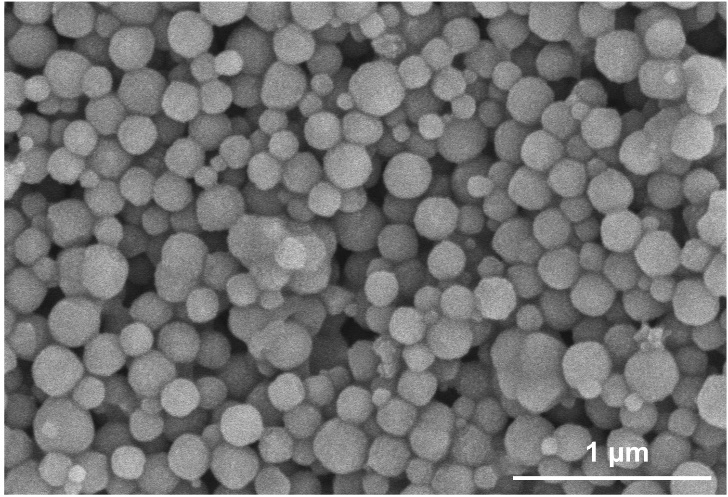
**

**Fig. S1** SEM image of FeS_2_ NPs, scale bar = 1 μm.


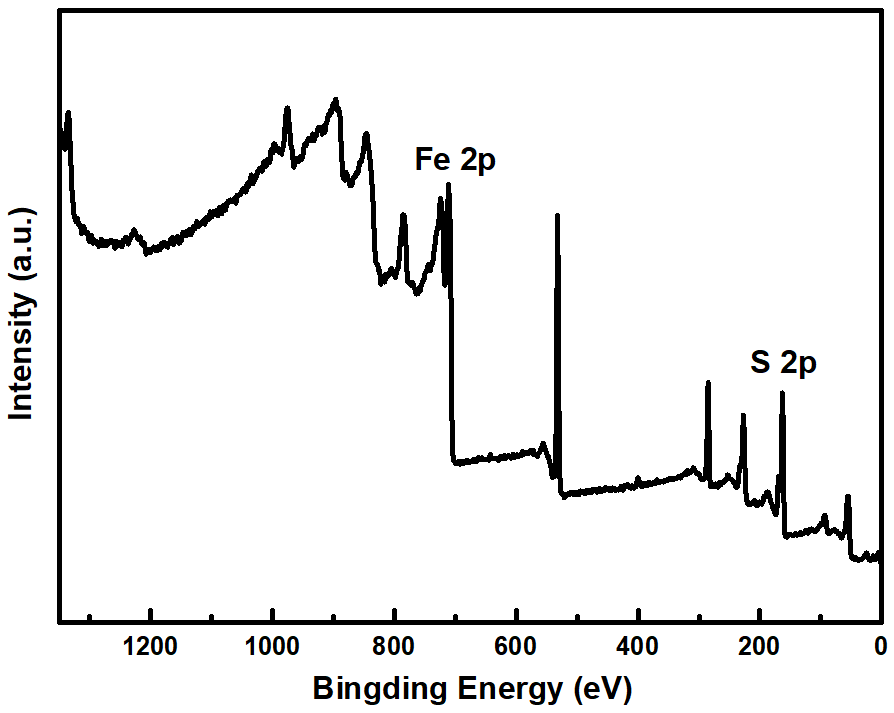


**Figure S2.** Survey XPS spectrum of FeS_2_ NPs


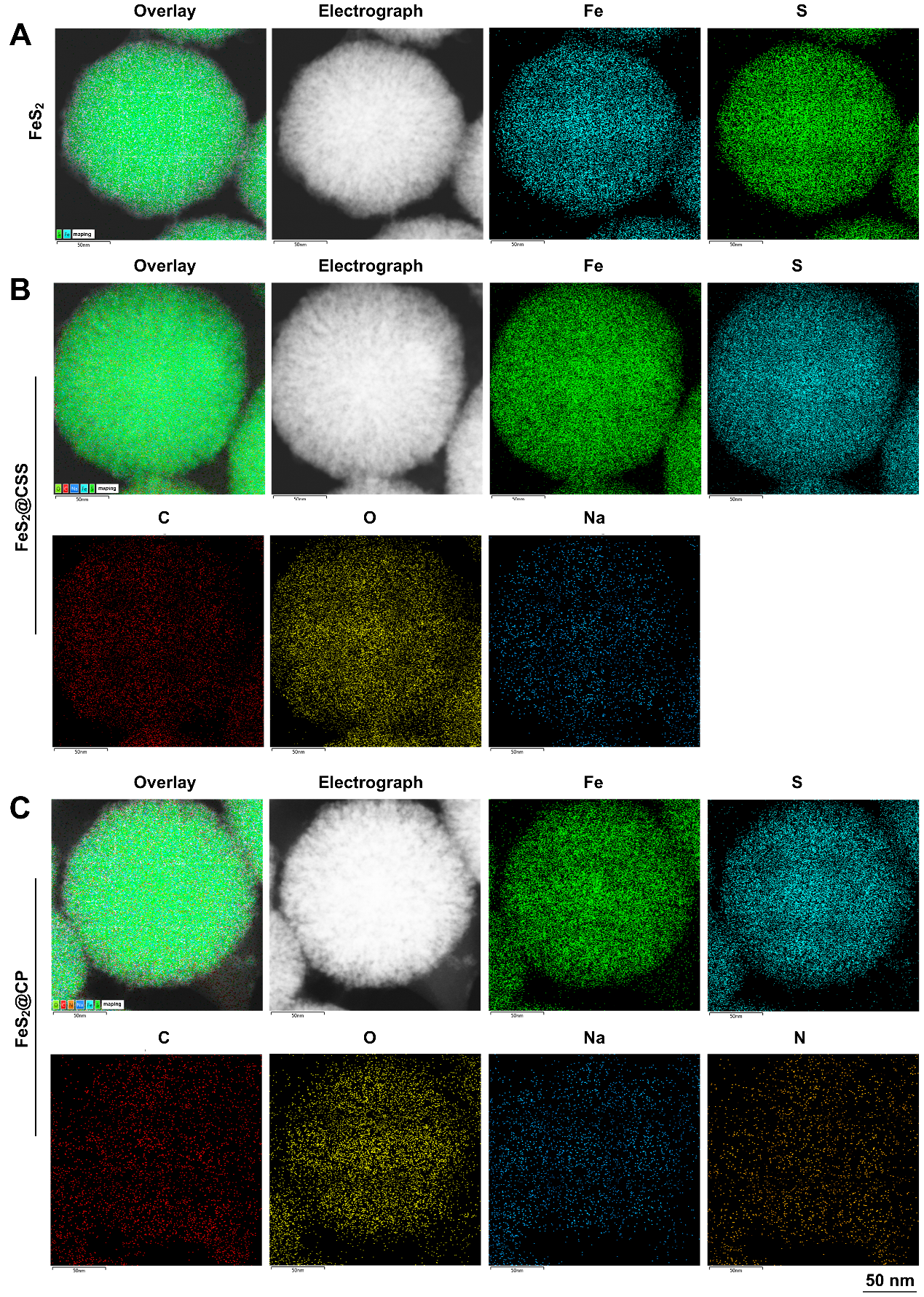


**Figure S3.** EDX-elemental mapping of (A) FeS_2_ NPs, (B) FeS_2_@CSS NPs and (C) FeS_2_@CP NPs, scale bar = 50 nm.

**Table S1.** Chemical compositions of FeS_2_ NPs, FeS_2_@CSS NPs and FeS_2_@CP NPs via EDX analysis.

| **Sample**  **Element** | **k-line factor** | **Entry** | | | | | |
| --- | --- | --- | --- | --- | --- | --- | --- |
|  |  | **FeS_2_** | | **FeS_2_@CSS** | | **FeS_2_@CP** | |
|  |  | **wt %** | **atom %** | **wt %** | **atom %** | **wt %** | **atom %** |
| **S** | 0.980 | 56.32 | 69.19 | 42.03 | 38.41 | 38.97 | 35.14 |
| **Fe** | 1.214 | 43.68 | 30.81 | 37.84 | 19.91 | 39.51 | 20.47 |
| **C** | 3.115 | - | - | 8.2 | 20.01 | 9.36 | 22.53 |
| **O** | 1.455 | - | - | 9.96 | 19.82 | 8.75 | 15.81 |
| **Na** | 1.124 | - | - | 1.97 | 1.85 | 1.21 | 1.52 |
| **N** | 1.807 | - | - | - | - | 2.2 | 4.53 |

**
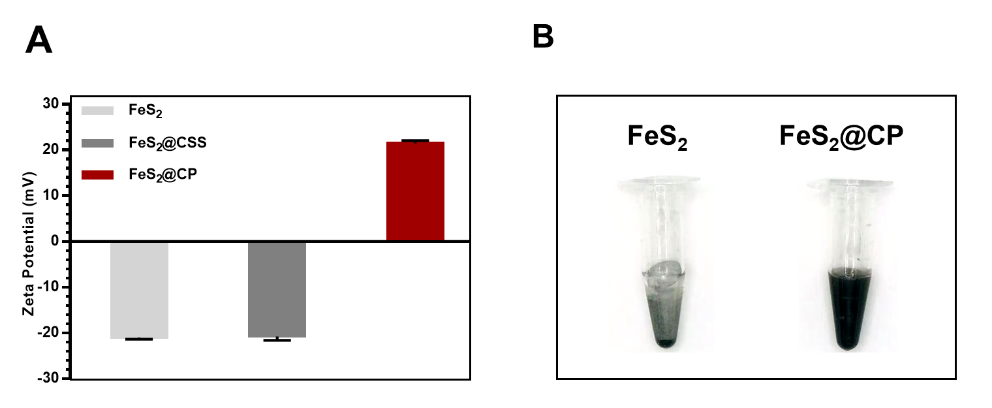
**

**Fig. S4** (A) Zeta potential values of FeS_2_-based NPs tested by DLS. All data were expressed as the mean ± SD, n = 3. (B) Precipitation of FeS_2_-based NPs in aqueous solution after overnight standing.


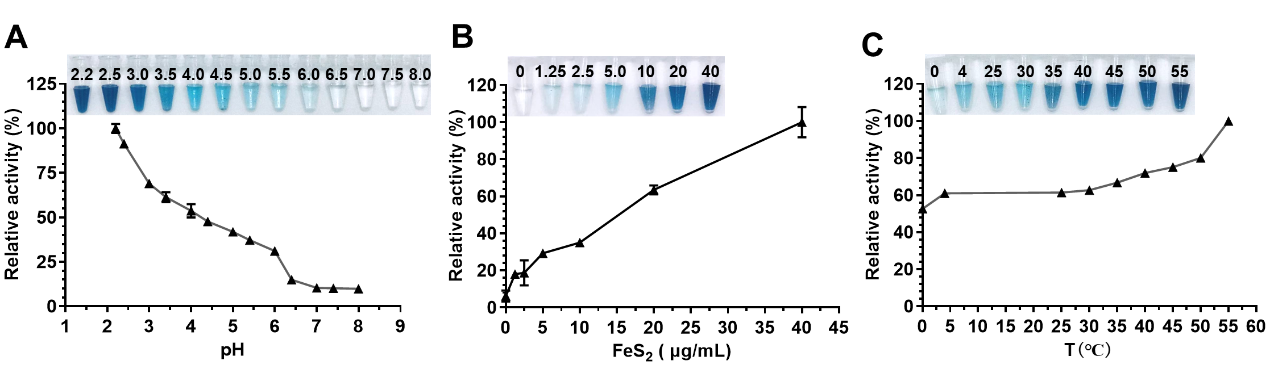


**Fig. S5** The catalytic activity of FeS_2_ NPs as Fenton reagents. (A) The concentration-, (B) pH-, and (C) temperature-dependent Fenton catalytic activity of FeS_2_ NPs with H_2_O_2_ substrate. All data were expressed as the mean ± SD (n = 3. Statistics were done using one-way ANOVA with Tukey multi-comparisons. *p < 0.05, **p < 0.01, ***p < 0.001 and ns, no significance).

**
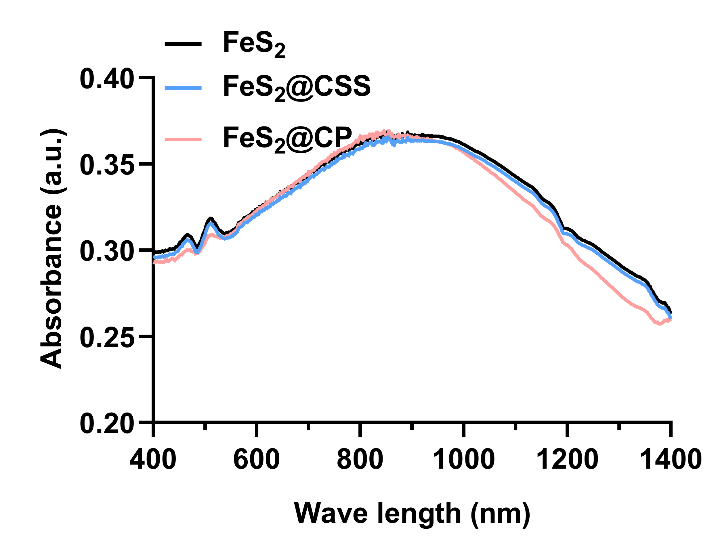
**

**Fig. S6** UV-Vis absorption spectrum for FeS_2_ NPs, FeS_2_@CSS NPs and FeS_2_@CP NPs.

**
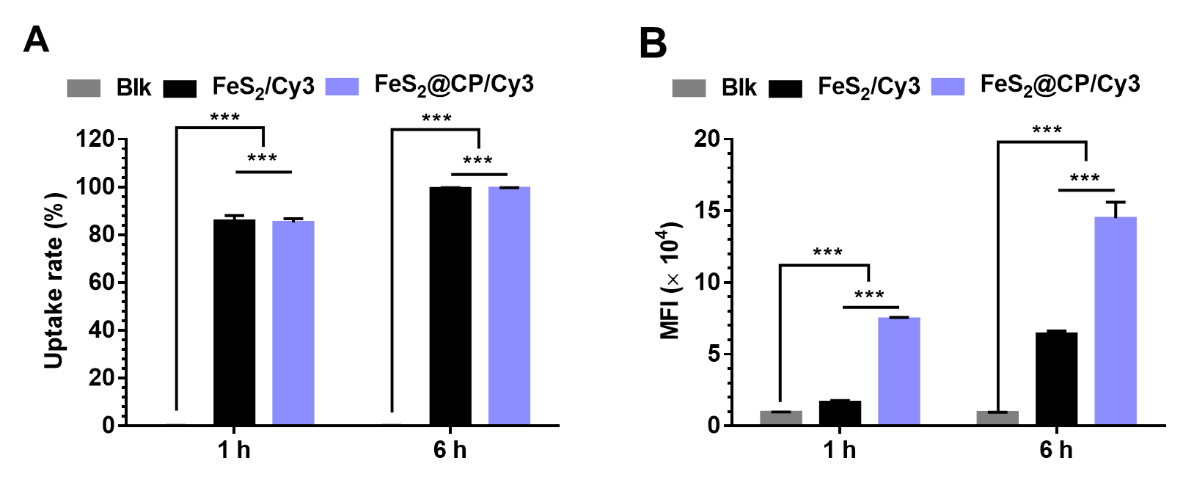
**

**Fig. S7** (A) Quantitative analysis of cell uptake rates and (B) the mean fluorescence intensity (MFI) analysis on U2-OS cells treated with FeS_2_-based NPs detected by flow cytometry. Blk: Blank. All data were expressed as the mean ± SD (n = 3. Statistics were done using one-way ANOVA with Tukey multi-comparisons. *p < 0.05, **p < 0.01, ***p < 0.001 and ns, no significance).


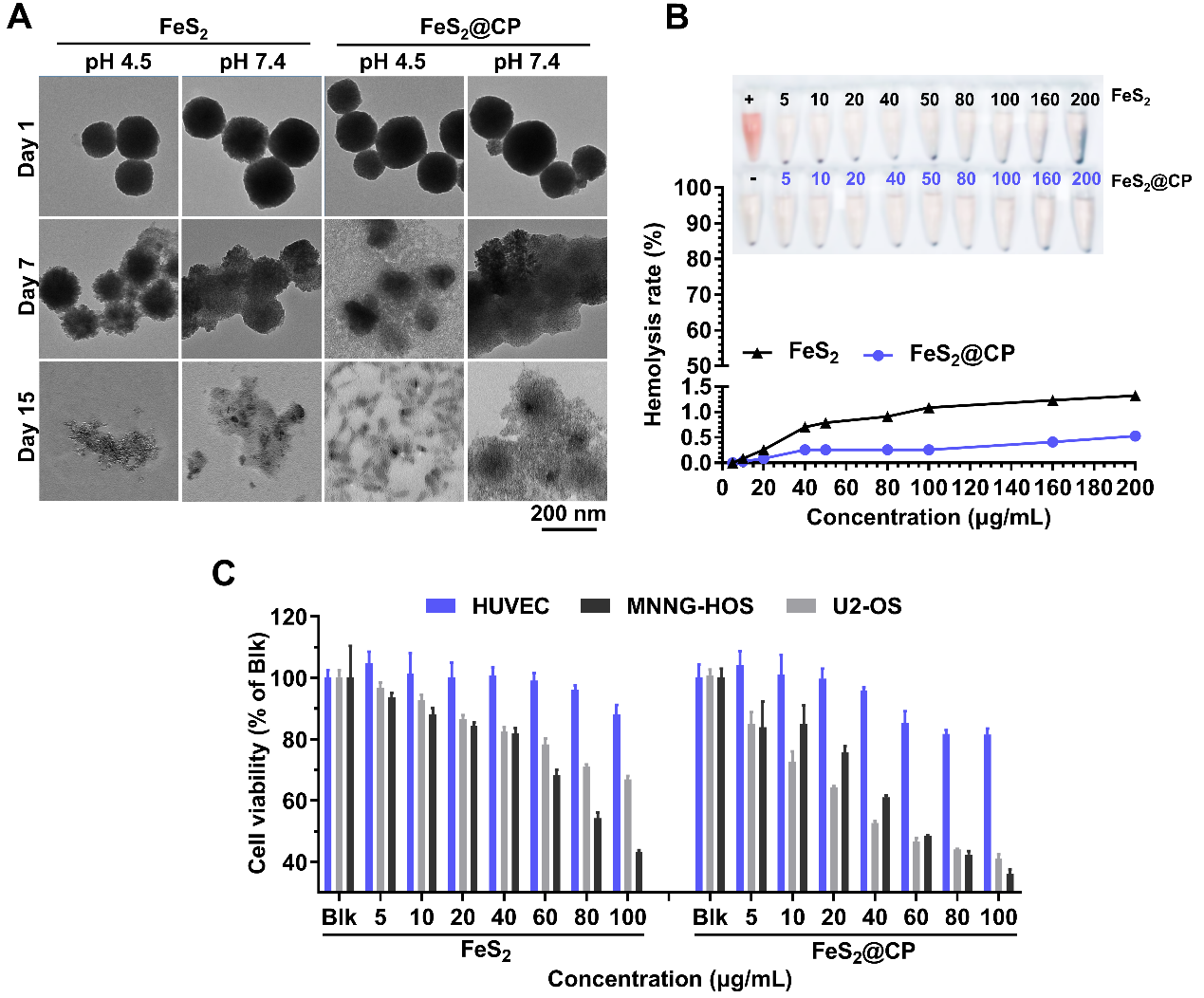


**Fig. S8** (A) Biodegradation of FeS_2_-based NPs in pH 4.5 and pH 7.4 buffer (mimicking lysosome and cytoplasm environment, respectively). (B) Hemolysis assay of FeS_2_-based NPs with different concentrations. (C) Cytotoxicity of FeS_2_-based NP on osteosarcoma cell lines (MNNG-HOS and U2-OS) and normal cell line HUVEC. All data were expressed as the mean ± SD, n = 3.

**
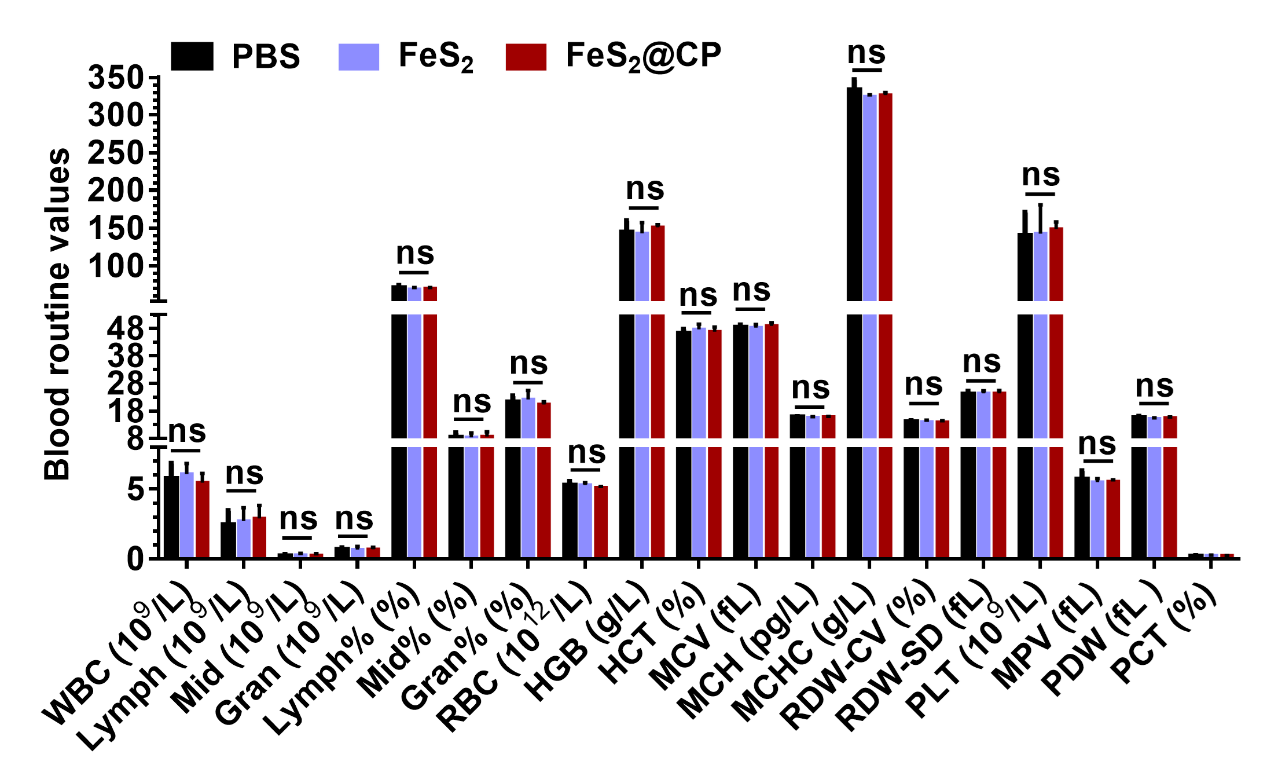
**

**Fig. S9** Routine blood values analysis for the BALB/c mice treated with PBS and different FeS_2_-based NPs for 15 days. WBC: white blood cell count, Lymph: lymphocyte count, Mid: intermediate cell count, Gran: granulose count, Lymph %: percentage of lymphocytes, Mid %: percentage of intermediate cells, Gran %: percentage of granulose, RBC: red blood cell count, HGB: Hemoglobin, HCT: hematocrit, MCV: mean corpuscular volume, MCH: mean corpuscular hemoglobin, MCHC: mean corpuscular hemoglobin concentration, RDW-CV: coefficient of variation of red cell distribution width, RDW-SD: red cell distribution width, PLT: platelet count, MPV: mean platelet volume, PDW: platelet distribution width, PCT: plateletcrit. All data were expressed as the mean ± SD (n = 4, one-way ANOVA and Tukey multi-comparisons. ns, no significance).

**
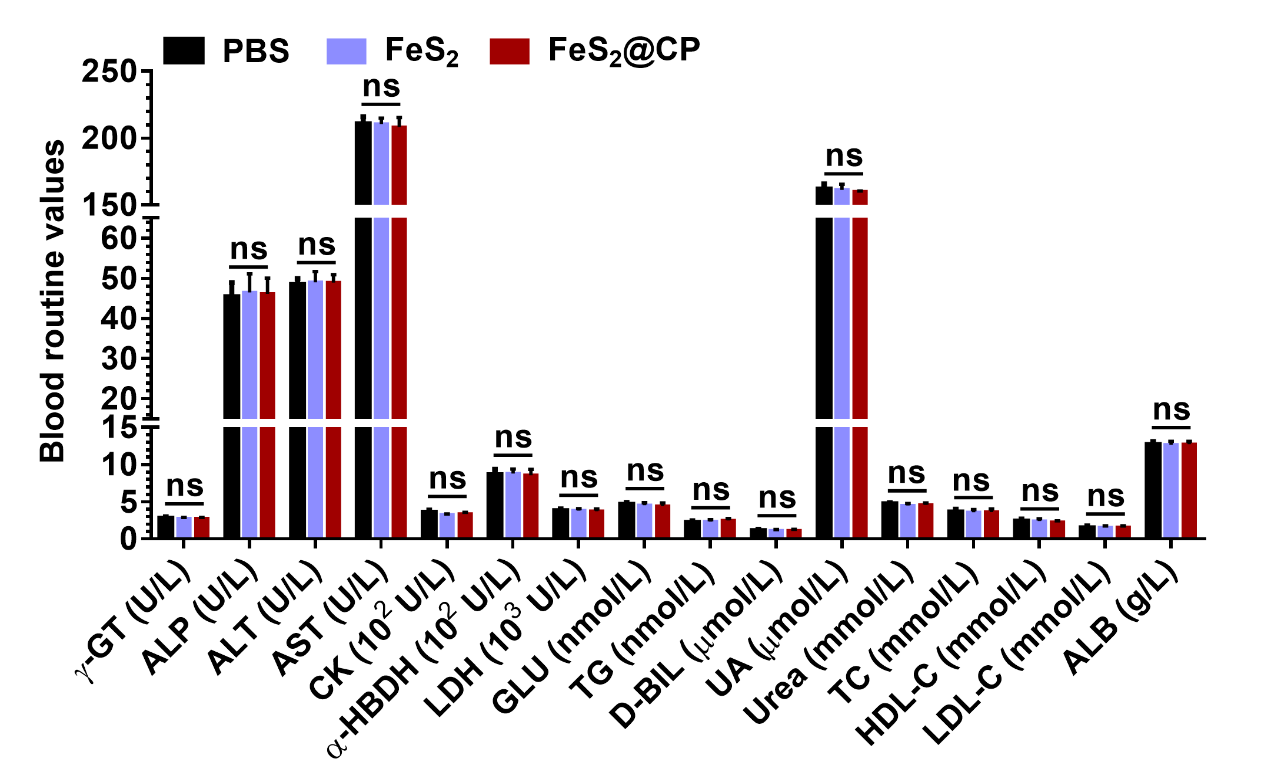
Fig. S10** Blood biochemistry analysis for the BALB/c mice treated with PBS and different FeS_2_-based NPs for 14 days. γ-GT: gamma-glutamyl transferase, ALP: alkaline phosphatase, ALT: alanine transaminase, AST: aspartate transaminase, CK: creatine kinase, α-HBDH: α-hydroxybutyrate dhehydrogenase, LDH: lactate dehydrogenase, GLU: blood glucose, TG: triglyceride, D-BIL: direct bilirubin, UA: uric acid, TC: total cholesterol, HDL-C: high-density lipoprotein cholesterol, LDL-C: low-density lipoprotein cholesterol, ALB: albumin. All data were expressed as the mean ± SD (n = 4, one-way ANOVA and Tukey multi-comparisons. ns, no significance).

**
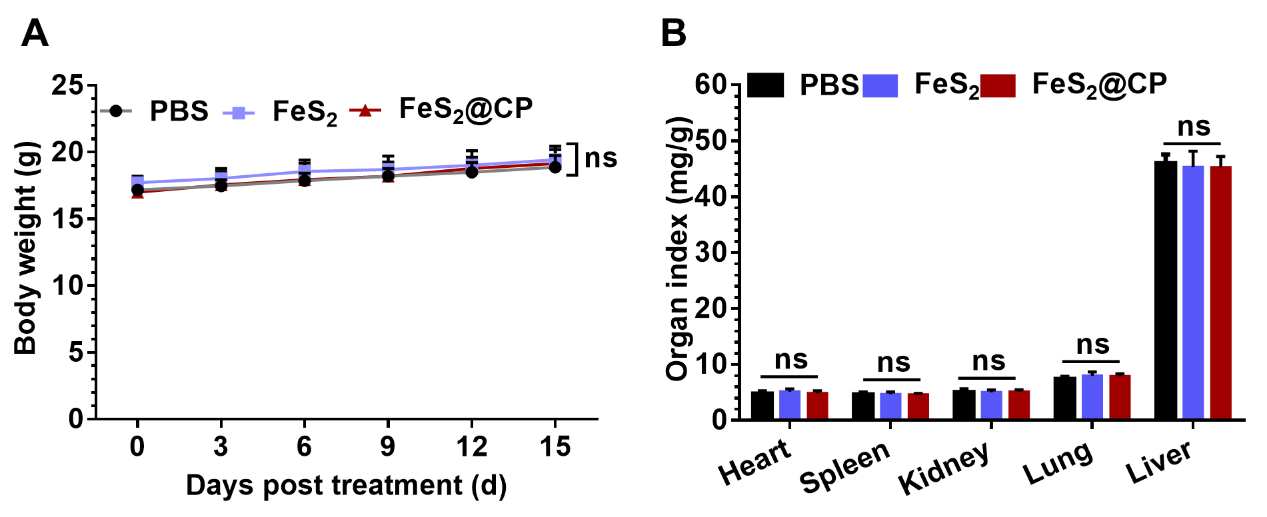
Fig. S11** (A) Bodyweight changes of the BALB/c mice during a 15-day treatment of PBS and different FeS_2_-based NPs. (B) Major organ indexes of mice in each group at treatment endpoint. All data were expressed as the mean ± SD (n = 4, one-way ANOVA with Tukey multi-comparisons. ns, no significance).

**
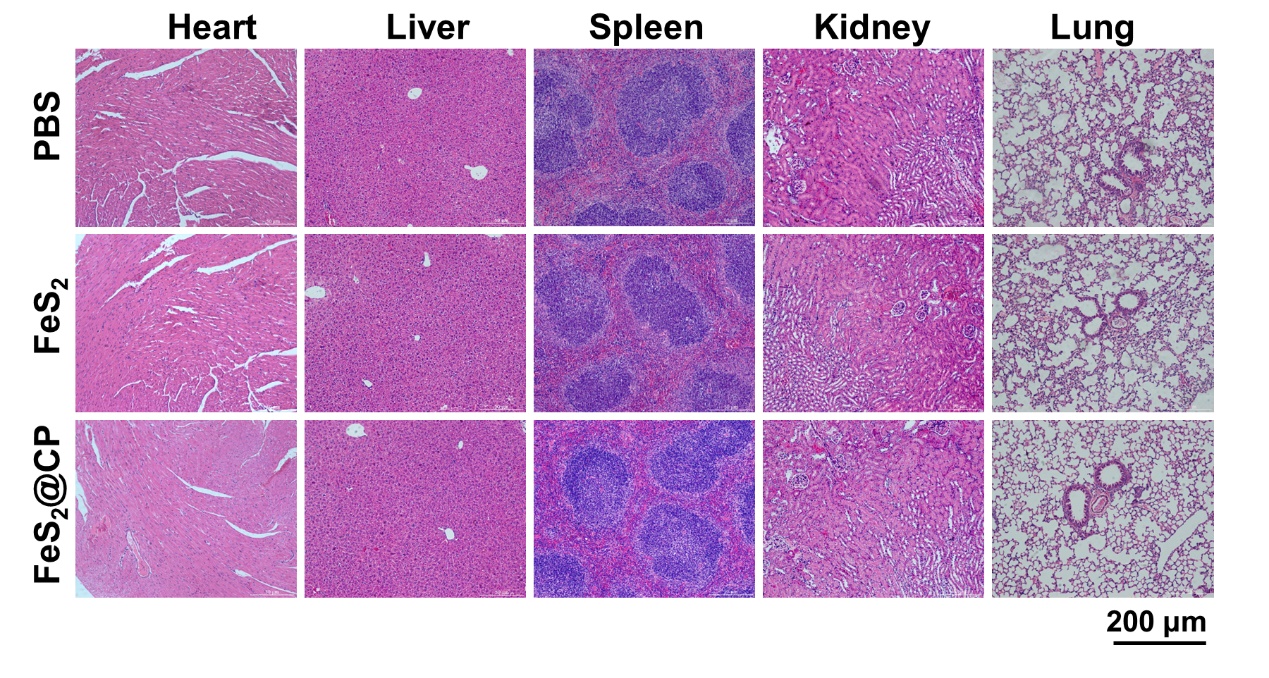
Fig. S12** Representative microphotographs of H&E staining of the sections from the major organs collected from the BALB/c mice at the treatment endpoint. Scale bar = 200 μm.

**
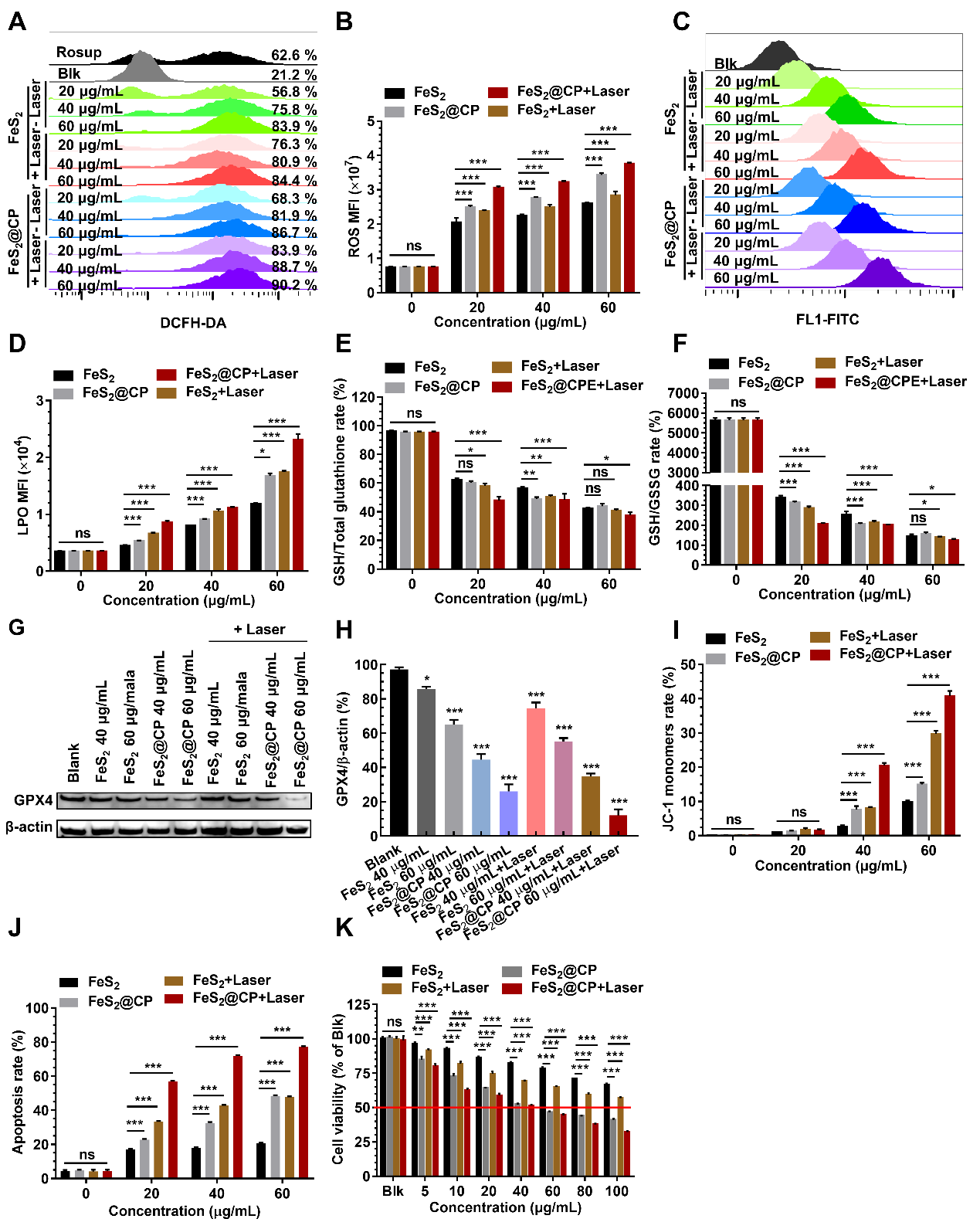
Fig. S13** Anti-cancer efficacy of FeS_2_-based NPs in U2-OS cells. Intracellular ROS level assessed with (A) the percentages of cells producing ROS and (B) the MFI of ROS detected by flow cytometry with DCFH-DA probe in U2-OS cells after FeS_2_-based NPs treatment for 24 h. (C) Intracellular LPO level detected by flow cytometry with C11-BODIPY 581/591 in U2-OS cells after FeS_2_-based NPs treatment for 24 h. (D) Quantitative MFI analysis of LPO recorded in panel (C). Loss of intracellular GSH assessed with (E) the rate of GSH/total glutathione and (F) the rate of GSH/GSSG in U2-OS cells after FeS_2_-based NPs treatment for 24 h. (G) Western blot analysis of GPX4 expression in U2-OS cells treated with different nano-formulations for 24 h with or without laser irradiation, and (H) GPX4 protein expression was quantified and normalized with β-actin. (I) Quantitative analysis of JC-1 monomers rate to assess the mitochondrial membrane potential of U2-OS cells induced by FeS_2_-based NPs treatment for 24 hours. (J) Quantitative analysis U2-OS cells apoptosis induced by FeS_2_-based NPs treatment for 24 hours. (K) Cell viability of U2-OS cells was analyzed by a CCK-8 assay after the treatment with FeS_2_-based NPs for 24 h. All data were expressed as the mean ± SD (n = 3. Statistics were done using one-way ANOVA with Tukey multi-comparisons. *p < 0.05, **p < 0.01, ***p < 0.001 and ns, no significance).


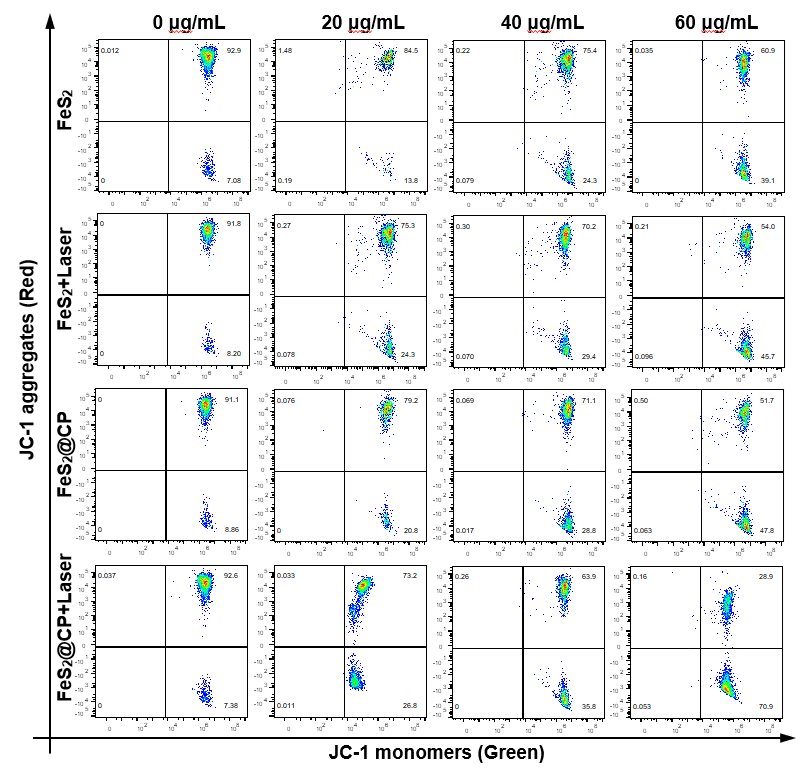


**Fig. S14** Flow cytometry detection of mitochondrial membrane potential with JC-1 probe on MNNG-HOS cells treated with different concentrations of FeS_2_-based NPs for 24 h.


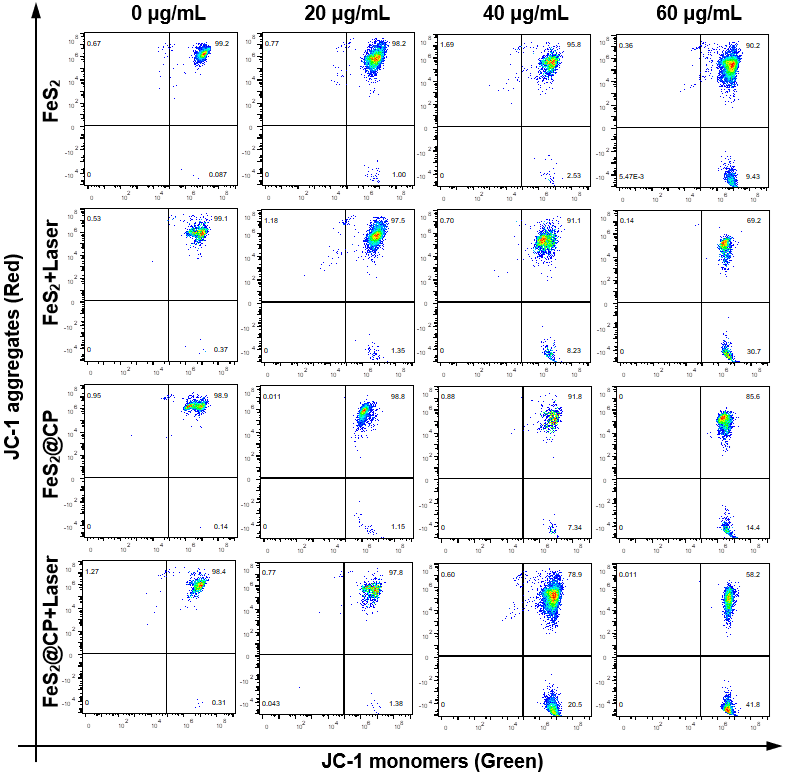


**Fig. S15** Flow cytometry detection of mitochondrial membrane potential with JC-1 probe on U2-OS cells treated with different concentrations of FeS_2_-based NPs for 24 h.


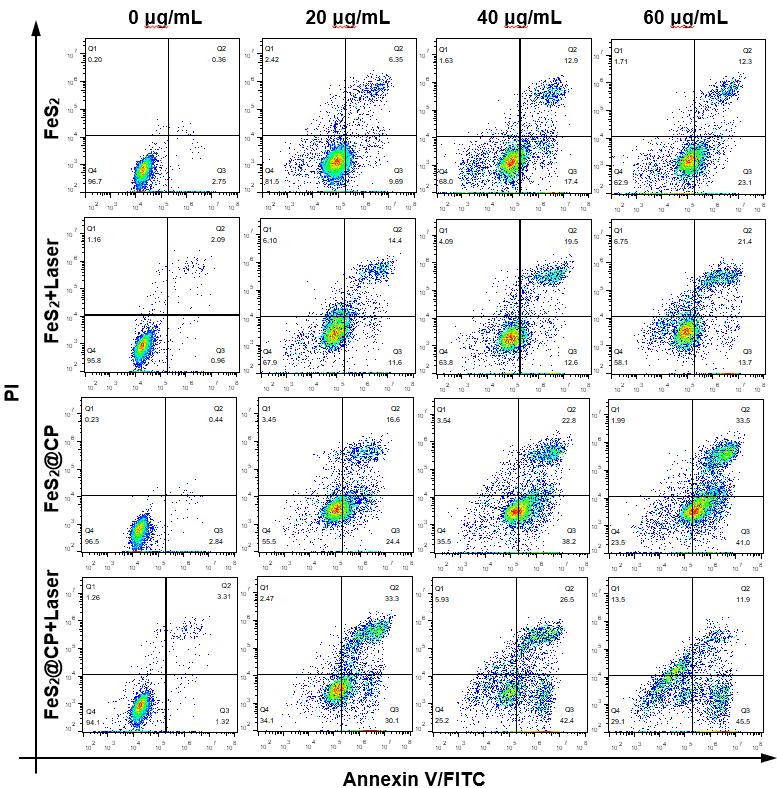


**Fig. S16** Flow cytometry detection of apoptosis with Annexin V/FITC and PI on MNNG-HOS cells treated with different concentrations of FeS_2_-based NPs for 24 h.

**
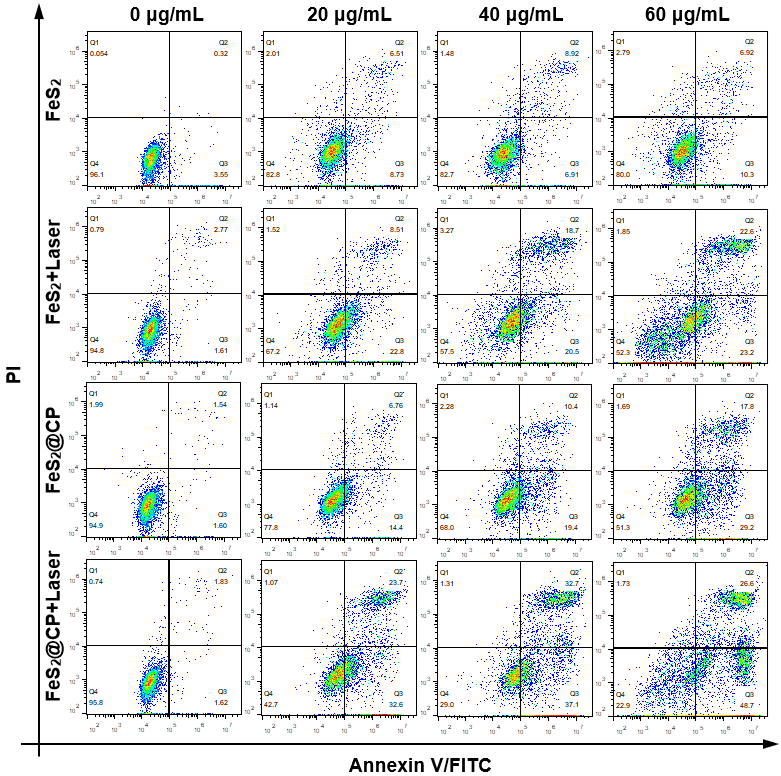
**

**Fig. S17** Flow cytometry detection of apoptosis with Annexin V/FITC and PI on U2-OS cells treated with different concentrations of FeS_2_-based NPs for 24 h.

**
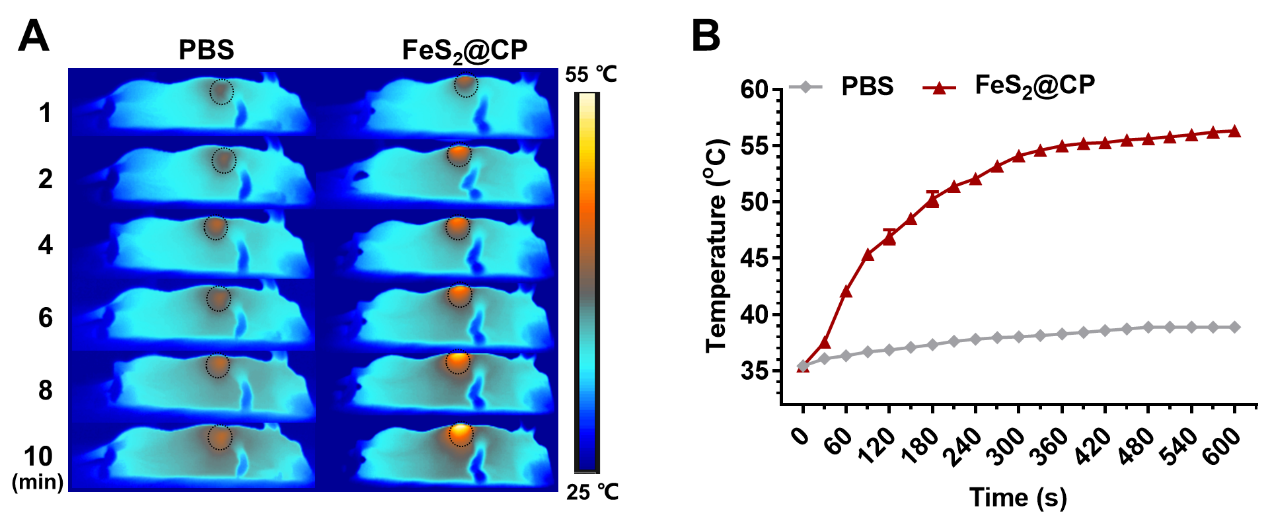
Fig. S18** (A) In vivo photothermal images of MNNG-HOS tumor-bearing mice intratumorally injected with PBS and different FeS_2_-based NPs dispersion activated by 1 W/cm^2^ 1064 nm laser for 10 min. The dotted circle represents the tumor site. (B) The temperature increased curve of tumor area under laser irradiation, n = 3.

**
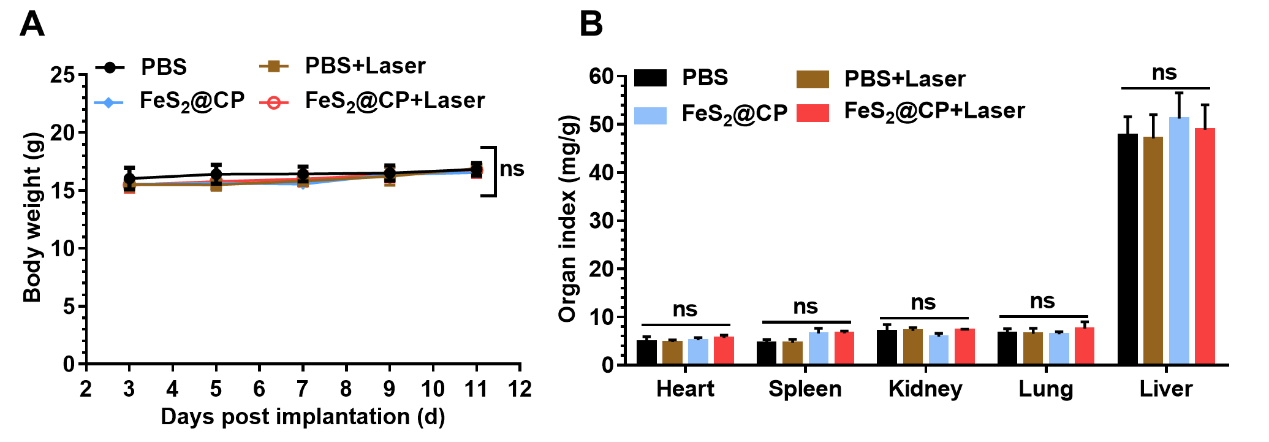
Fig. S19.** (A) Bodyweight changes of the MNNG-HOS tumor-bearing BALB/c nude mice during the treatment of the test groups (B) Main organ index of mice in each test group at treatment endpoint. All data were expressed as the mean ± SD (n = 5, one-way ANOVA with Tukey multi-comparisons. ns, no significance).

**
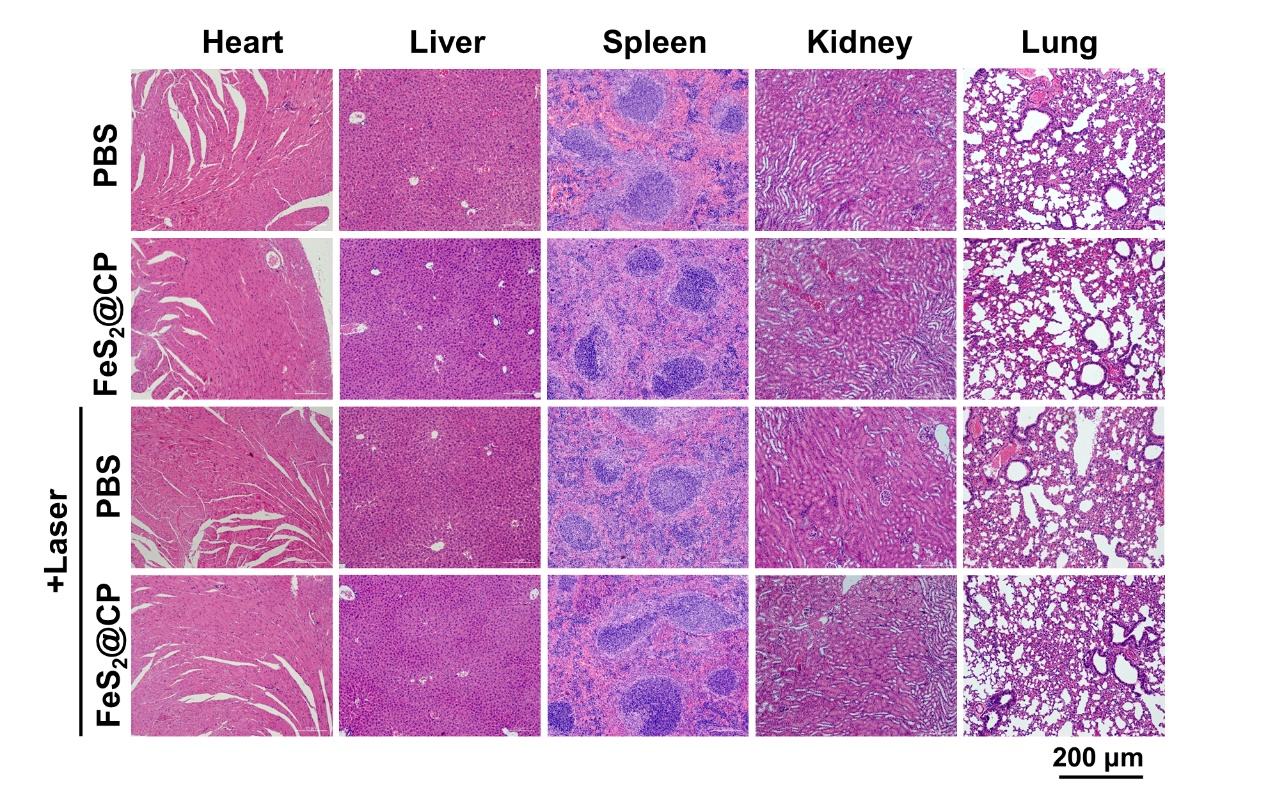
Fig. S20.** Representative microphotographs of H&E staining of the sections from the major organs collected from the MNNG-HOS tumor-bearing BALB/c nude mice in different groups at the treatment endpoint. Scale bar = 200 μm.
